# Meta-analysis of NAD(P)(H) quantification results exhibits variability across mammalian tissues

**DOI:** 10.1038/s41598-023-29607-8

**Published:** 2023-02-11

**Authors:** Dassine Azouaoui, Michael René Choinière, Momtafin Khan, Shahab Sayfi, Simran Jaffer, Selvia Yousef, David A. Patten, Alexander E. Green, Keir J. Menzies

**Affiliations:** 1 Department of Biochemistry, Microbiology and Immunology, Faculty of Medicine, University of Ottawa, Ottawa, ON Canada; 2 Ottawa Institute of Systems Biology, University of Ottawa, Ottawa, ON Canada; 3 Interdisciplinary School of Health Sciences, Faculty of Health Sciences, University of Ottawa, Ottawa, ON Canada

**Keywords:** Diagnostic markers, Metabolomics

## Abstract

Nicotinamide Adenine Dinucleotide (NAD^+^) plays an important role in energy metabolism and signaling pathways controlling crucial cellular functions. The increased interest in NAD^+^ metabolism and NAD^+^-boosting therapies has reinforced the necessity for accurate NAD^+^ quantification. To examine the published NAD(P)(H) measures across mammalian tissues, we performed a meta-analysis of the existing data. An Ovid MEDLINE database search identified articles with NAD(P)(H) quantification results obtained from mammalian tissues published between 1961 and 2021. We screened 4890 records and extracted quantitative data, as well as the quantification methods, pre-analytical conditions, and subject characteristics. The extracted physiological NAD(P)(H) concentrations in various tissues from mice, rats, and humans, revealed an important inter- and intra-method variability that extended to recent publications. This highlights the relatively poor potential for cross-experimental analyses for NAD(P)(H) quantitative data and the importance of standardization for NAD(P)(H) quantification methods and pre-analytical procedures for future preclinical and clinical studies.

## Introduction

Nicotinamide adenine dinucleotide plays a dual role as a reduction–oxidation (redox) co-enzyme, in its oxidized (NAD(P)^+^) and reduced (NAD(P)H) forms, and as a co-substrate (NAD(P)^+^) for enzymes involved in deacylation^1^, mono- and poly-ADP-ribosylation^2,3^, and the creation of adenine-based second messengers^4–7^. The NAD(P)^+^/NAD(P)H ratio is an important indicator of the intracellular redox state and plays a critical role in the regulation of key metabolic pathways^8,9^. For example, NAD^+^ is essential for glycolysis, along with the tricarboxylic acid (TCA) cycle, to provide reducing equivalents during the generation of adenosine triphosphate (ATP), the main source of energy for the cell^8,9^. When NAD^+^ is limited it can also be regenerated from NADH by the fermentation of pyruvate to produce lactate in mammalian cells, thereby maintaining cellular redox balance^25^. NAD^+^ and NADH can also be phosphorylated to NADP and NADPH, respectively, by NAD^+^ Kinases (NADKs)^10,11^. NADPH is essential for reductive biosynthesis, signal transduction, cellular antioxidant responses, and particularly, glutathione reduction^12–14^. In the cytosol, NADP^+^ is reduced to NADPH by the glucose-6-phosphate dehydrogenase and 6-phosphogluconate dehydrogenase of the pentose phsophate pathway (PPP), amongst other reactions^13^. Whereas in the mitochondria, NADPH production is sustained by various reactions, including those reliant on nicotinamide nucleotide transhydrogenase (NNT) and isocitrate dehygronases (IDH2) enzyme activity^15^. As a result, NAD(P)(H) status has been shown to affect cell signaling and metabolism, energy homeostasis, mitochondrial biogenesis, oxidative stress responses and DNA maintenance and repair^16–18^. Further, since the progression of age-related diseases, such as obesity^19^, non-alcoholic fatty liver disease^20,21^, neurodegeneration^22^, mitochondrial^23^ and cardiovascular^24^ diseases, and muscle atrophy or dystrophy^25^, has been linked to alterations in NAD^+^-related metabolites, approaches aimed at maintaining NAD^+^ homeostasis are currently under investigation.

Beyond common redox transitions, NADP^+^ can be consumed by exchanging its nicotinamide group with nicotinic acid in a reaction catalyzed by cADPR synthases, such as CD38, to form the second messenger NAADP, which is involved in calcium mobilization, hormone secretion, and immune cell proliferation^6,7,26^. NAD^+^ can also be consumed when acting as a co-substrate to form nicotinamide (NAM), which can be used as a substrate to regenerate NAD^+^ via the NAM salvage pathway. NAM can also be methylated to form 1-methyl NAM (meNAM), which can be further oxidized to the degradation products N1-methyl-2-pyridone-5-carboxamide (2py) and N1-methyl-4-pyridone-3-carboxamide (4py). Ultimately, the methylated catabolites can be cleared from the body via urine^27,28^. The NAD^+^ pool can also be reduced by consumption of NADP^+^ via a base-exchange reaction for the formation of the second messenger NAADP^14^. Maintaining the pool of NAD^+^ therefore requires biosynthesis via the Preiss-Handler pathway (starting with the common vitamin B3 molecule niacin (NA; nicotinic acid)), the de novo biosynthesis pathway (starting with the amino acid tryptophan (Trp)) or regeneration from the conversion of NAM via the salvage pathway to NMN then to NAD^+^. Finally, the salvage pathway is also important for the biosynthesis of NAD^+^ from nicotinamide riboside (NR), an alternative form of vitamin B3 found in foods, following its phosphorylation by nicotinamide riboside kinase to NMN and then conversion to NAD^+^ via the salvage pathway.

Understanding the dynamics of the NAD^+^ metabolome has therefore become essential and ultimately requires rigorous metabolite quantification in order to better understand the flux of NAD^+^ metabolites during normal physiology, or with disease progression or healthy aging. A clear picture of these changes is essential for linking alterations in the NAD^+^ metabolome to downstream enzymatic or redox reactions, which hold the key to changes in tissue phenotypes. Given the substantial amount of previously reported quantitative data on NAD^+^ metabolites in mammals, including a growing number of reports in humans, it is essential and timely to summarize these changes during health and disease. Numerous studies have examined components of the NAD^+^ metabolome in rodents and humans under different metabolic states, ages, and diseases, yet rely on different methods for sample preparation or quantification. Sample preparation is important for metabolites involved in redox reactions due to potential degradation and inter-conversions under certain conditions^29,30^. For quantification, liquid chromatography coupled to mass spectrometry (LC–MS) and magnetic resonance spectrometry (MRS) are thought to be sensitive and specific methods^31–33^, while ezyme cycling assays are the more common. Quantitative LC–MS methods require a comprehensive inclusion of quality controls for individual metabolites being measured and for the overall matrix effect of the samples, an effect that explains varying mass spectrometric responses for a given analyte when measured in different solutions. Without these controls, LC–MS is at best semi-quantitative. In this study, we identified considerable variability in NAD(P)(H) measures across rodent and human studies, emphasizing the need for proper methods reporting and for standardized sample processing and analytical protocols. Overall, comparable NAD(P)(H) datasets across studies is needed for meaningful interpretation of the existing and future data in the field of NAD^+^ biology.

This study provides (1) a meta-analysis of the observed physiological NAD(P)(H) metabolite levels, in different tissues from mice, rats, and humans; (2) a summary of the effects of tissue sampling conditions and analytical procedures on NAD(P)(H) measurements in mammalian tissues; and (3) highlights of crucial steps in the pre-analytical and analytical procedures that need to be optimized.

## Results

### Meta-analysis of common quantification methods, along with species, sex, and tissues studied

Historically, early methods used for NAD(P)(H) quantification relied on the spectrophotometric or fluorometric properties of these metabolites^34,35^. More recently, enzyme cycling methods coupled to spectrophotometric or fluorometric detection are more commonly used to distinguish NAD(P)(H) co-enzyme levels^36–38^. The growing need for high sensitivity and resolution in the quantification of these co-enzymes, along with the necessity to measure other NAD^+^-related metabolites, led to the use of high-performance liquid chromatography (HPLC) and LC–MS techniques. Of these approaches, LC–MS has become the preferred method, as mass spectrometry combined with liquid chromatography allows for the specific identification of eluted compounds along with the added benefit for including NAD(P)(H) isotope metabolites as internal spike-in quality controls. Despite their advantages over classic spectrophotometric or fluorometric assays, HPLC and LC–MS both remain expensive and time consuming, which explains why enzyme cycling assays persist in recent studies.

**Figure 1:**
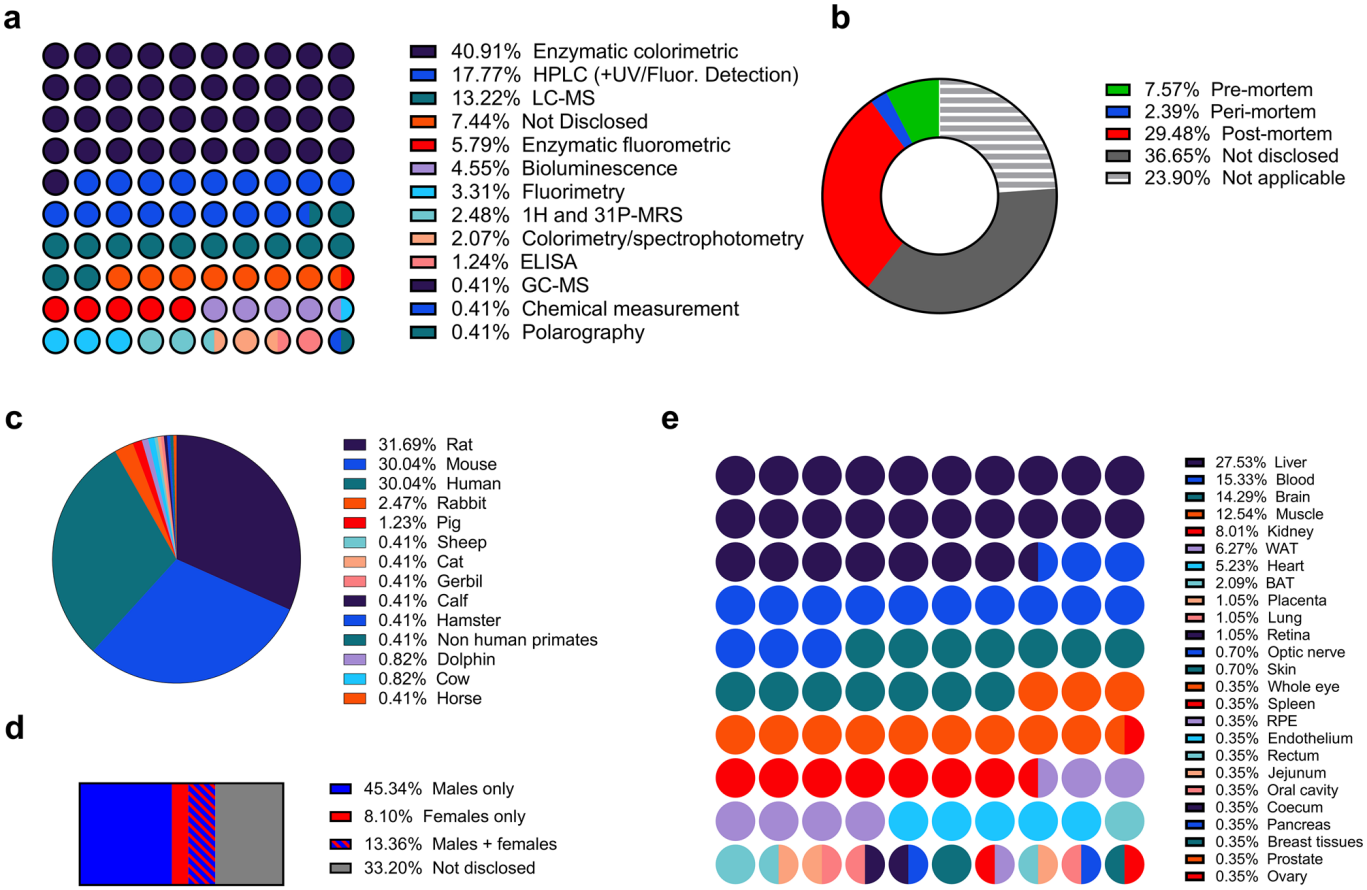
Methodological characteristics of included studies. Distribution of (**a**) NAD(P)(H) quantification methods, (**b**) sampling timepoint relative to sacrifice, (**c**) mammalian species, (**d**) sex, and (**e**) tissues used among eligible studies. *WAT* white adipose tissue, *BAT* brown adipose tissue, *RPE* retinal pigment epithelium.

Using a systematic approach, of the 241 eligible studies (Supplementary Material 2), 205 studies that included quantitative meta-analysis of NAD(P)(H) concentrations in mouse, rat, and human tissues were selected for further analysis. Of these studies, 46.7% used enzyme cycling assays, of which included colorimetric (40.9%) or fluorometric (5.8%) detection, while 17.8% used HPLC methods (coupled with UV or fluorescence detectors) and 13.2% used LC–MS assays (Fig. 1a). Most studies did not include a detailed description of the metabolite extraction procedures. However, commonly used extraction procedures consisted of either extraction buffers with or without surfactants (Triton®, Tris, Dodecyltrimethylammonium bromide), or polar organic solvents such as acetonitrile, methanol, ethanol, or chloroform. Previous studies have shown that enzyme inactivation and optimal pH and temperature conditions were efficient at preserving the stability of the different NAD(P)(H) forms^30,39,40^. When disclosed, samples were processed under low-temperature conditions and no redox additives were mentioned in the included studies. Redox reactions were limited by the inactivation of the enzymes in the samples prior to NAD(P)(H) metabolites measurements, usually by precipitation with solvents such as ethanol, methanol, acetonitrile, or perchloric acid (PCA). However, the use of PCA was rare due to the acid-labile nature of the reduced forms (i.e. NADH and NADPH), as well as the necessity of an additional neutralization step. Thus among the studies reporting the use of PCA during the metabolite extraction (5.4%), 3.7% reported NAD(P)^+^ results only or used a second non-acidic extraction method for proper NAD(P)H quantification. Only 1.7% of the studies reported NADH, total NAD(H), and/or NAD^+^/NADH results using only the PCA extraction. The potential bias from these studies could not be analyzed due to the limited data.

Additionally, within the included studies 36.65% did not disclose the time of tissue harvest relative to sacrifice, and when disclosed, the majority used post-mortem samples (29.48%) while only 7.57% used pre-mortem samples (Fig. 1b). Most of these studies were performed on rat (31.7%), mouse (30.0%) or human (30.0%) subjects and only 21.46% included females, with 33.2% of the studies not disclosing the sex of their subjects (Fig. 1c,d). Finally, 27.5% of the studies examined liver tissue, with the rest being predominantly focused on blood (15.3%), brain (14.3%), muscle (12.5%) and kidney (8.0%) (Fig. 1e).

### Comparisons of mean physiological NAD(P)(H) levels across tissues for rodents and humans

To help identify the expected mean physiological NAD(P)(H) levels for the most commonly studied mouse, rat and human tissues, we compiled data from all control and wild-type cohorts from each of the included studies (Supplementary Material 2). Specifically, we recorded the mean NAD(P)(H) levels normalized to tissue weight or, in the case of blood, volume. Data from studies that normalized metabolite levels to protein content were included in our compiled dataset (Supplementary Material 2) but were not used for further analysis given the low number of reported results. Additionally, when possible, we calculated the NAD(P)^+^/NAD(P)H ratios, as a reflection of the redox state, as well as the total NAD(P)(H) levels (NAD(P)^+^  + NAD(P)(H)) which are common reporting outputs for NAD(P)(H) fluxes. The extracted data also included the species, strains, age, sex, quantification method, diet and feeding schedule, sample type, and sample collection method and time of harvest (pre- or post-mortem).

**Figure 2:**
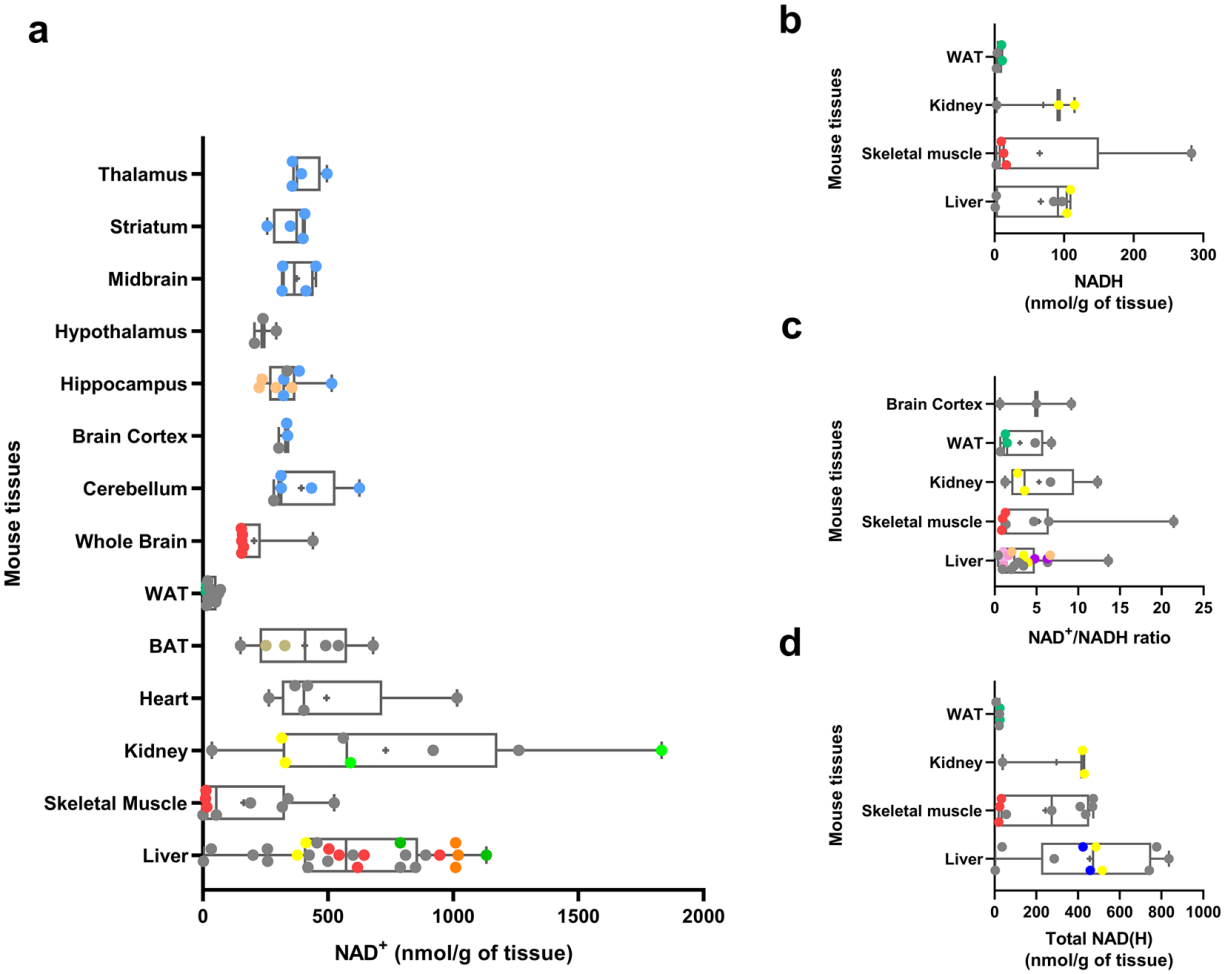
Reported physiological NAD^+^, NADH, total NAD(H) and NAD^+^/NADH ratio in normal mouse tissues with n ≥ 3. a-d: Reported mean (**a**) NAD^+^, (**b**) NADH, (**c**) NAD/NADH levels and (**d**) total NAD(H) in various mouse tissues (nmol/g of weight of tissue) collected from young (< 14 months old) control mice. The boxes represent the 25th to 75th percentiles with the median represented by the line inside the box. The means are shown as “ + ” symbol. The whiskers cover the minimum to maximum values. For each study that included more than one measurement per tissue, similar colors were assigned to the corresponding datapoints. Datapoints colored in grey represent studies with just one reported measurement for a given tissue. *WAT* white adipose tissue, *BAT* brown adipose tissue.

The meta-analysis of NAD(H) data identified the mean physiological concentrations for NAD^+^ and NADH in mouse (Fig. 2a,b, Supplementary Fig. 1a,b) and rat (Supplementary Fig. 2a,b) tissues, from animals younger than 14 months for mice and 18 months for rats. The extracted range of physiological NAD^+^/NADH ratio values and total NAD(H) levels (NAD^+^  + NADH) were plotted for mice (Fig. 2c,d) and rats (Supplementary Fig. 2c,d). Unlike the mouse studies, most of the reported rat data did not include the age of the animals; however, we chose to include these data provided that the study did not classify their cohorts as aged. Of the most prominently studied tissues, this meta-analysis shows that the median NAD^+^ level for liver (596 nmol/g, n = 26) is higher than all other tissues in mice, while surprisingly skeletal muscle (162.8 nmol/g, n = 9) exhibits one of the lowest median values per gram of tissue (Fig. 2a). These results may either imply a reduced efficiency in NAD^+^ metabolite extraction, due to the highly fibrous nature of muscle, or that there are important differences in muscle NAD^+^ metabolism when compared to other highly metabolic tissues, such as liver and kidney.

Despite few quantitative aging studies, NAD^+^ levels from aged mice were on average reduced in liver, muscle and retinal pigmented epithelium (RPE), when compared to their young cohorts, and in comparison to the mean of all studies performed with mice younger than 14 months of age (Supplementary Fig. 3). A meta-analysis of NADH and the NAD^+^/NADH ratio in aged animals was not possible given that only one of these quantitative studies performed these measurements. These data demonstrate the lack of quantitative NAD(H) data in aging animals.

**Figure 3:**
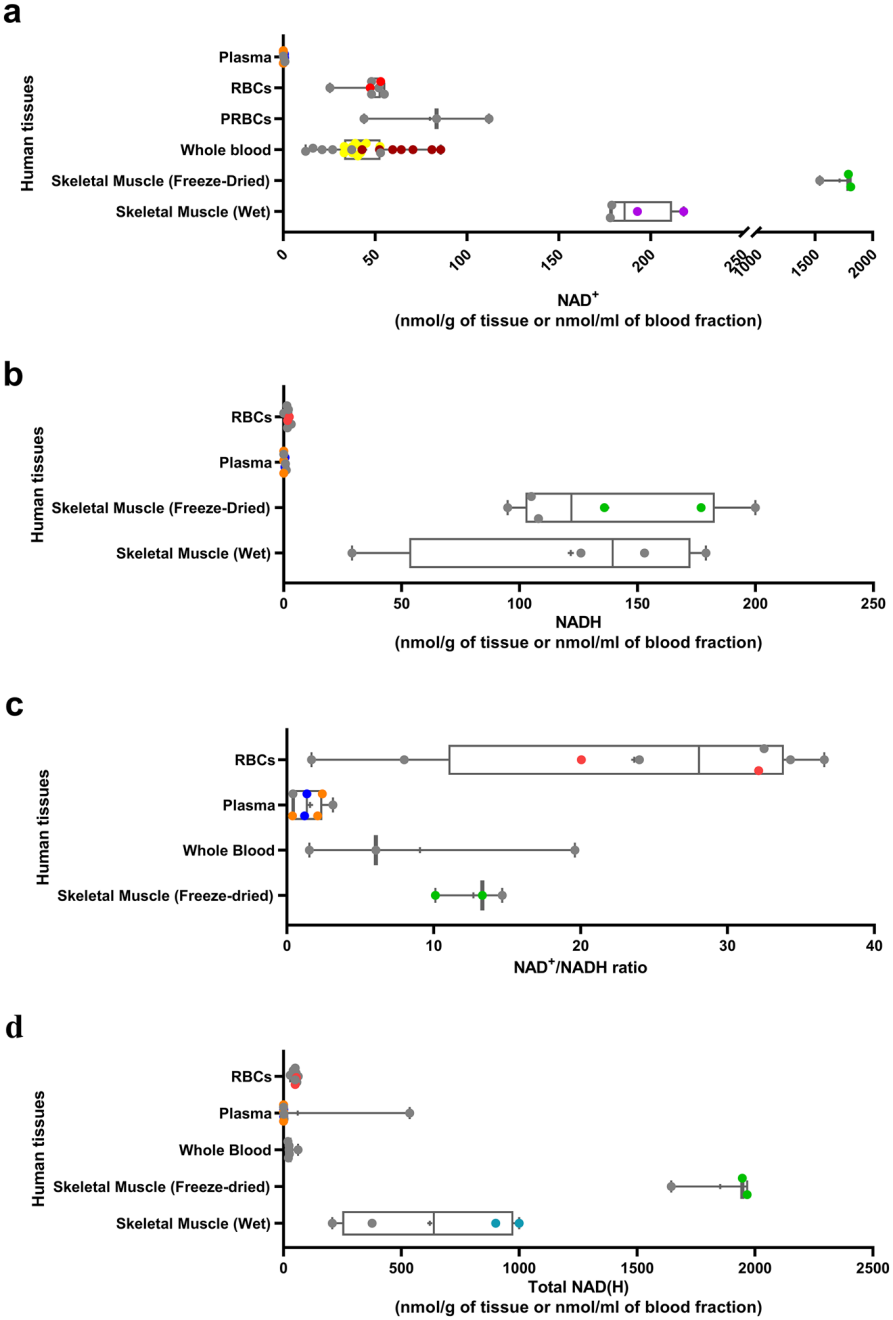
Reported physiological NAD^+^, NADH, total NAD(H) and NAD^+^/NADH ratio in human tissues with n ≥ 3. a-d: Reported mean (**a**) NAD^+^, (**b**) NADH, (**c**) NAD/NADH levels and (**d**) total NAD(H) in various human tissues (nmol/g of tissue weight or nmol/ml of blood fraction). The boxes represent the 25th to 75th percentiles with the median represented by the line inside the box. The means are shown as “ + ” symbol. The whiskers cover the minimum to maximum values. For each study that included more than one measurement per tissue, similar colors were assigned to the corresponding datapoints. Datapoints colored in grey represent studies with just one reported measurement for a given tissue. *PRBCs* packed red blood cells, *RBCs* red blood cells.

For the data retrieved from studies using healthy humans, the age ranges varied greatly across studies (from 15 to 92.3 years) and in most cases the data was not stratified across these ages. The most commonly measured human tissues included skeletal muscle and components of blood, due to their less invasive nature of collection (Fig. 3a–d). Results from other human tissues (n = 1–2) are displayed in Supplementary Fig. 4. This analysis exhibited a median NAD^+^ concentration of 44.62 nmol/ml (n = 23) in whole blood, with similar NAD^+^ concentrations reported for red blood cells at 46.96 nmol/ml (n = 7) (Fig. 3a; Supplementary Table 1). However, blood plasma NAD^+^ levels were substantially lower with a median of 0.37 nmol/ml (n = 8) (Fig. 3a). The median NAD^+^ levels found in skeletal muscle of humans was 191.9 (n = 4) nmol/g in wet muscle preparations versus 1713 (n = 3) nmol/g in freeze-dried preparations, a low temperature dehydration process that likely concentrates metabolites (Fig. 3a). In the more commonly investigated tissues for NADH measurements, the median levels in human red blood cells, plasma and freeze-dried skeletal muscle were 1.75 nmol/ml (n = 7), 0.39 nmol/ml (n = 8) and 136.8 nmol/g (n = 6), respectively (Fig. 3b). The corresponding median NAD^+^/NADH ratios were 23.65 (n = 8), 1.57 (n = 7) and 12.7 (n = 3) (Fig. 3c). Interestingly, the median NAD^+^/NADH ratio for red blood cells (23.65) and freeze-dried skeletal muscle (12.7) (Fig. 3c) were several fold higher than most mouse and rat tissues (Fig. 2c and Supplementary Fig. 2c), indicating a highly oxidative redox state with an abundance of available NAD^+^ in these human tissues. In the case of muscle tissue, this could have implications when considering the translational potential of rodent studies that have identified the benefits of boosting NAD^+^ levels in muscle. However, an elevated NAD^+^/NADH ratio in muscle may also be the result of delayed sample freezing procedures in a clinical environment, which may lead to non-physiological changes in sample redox status.

**Figure 4:**
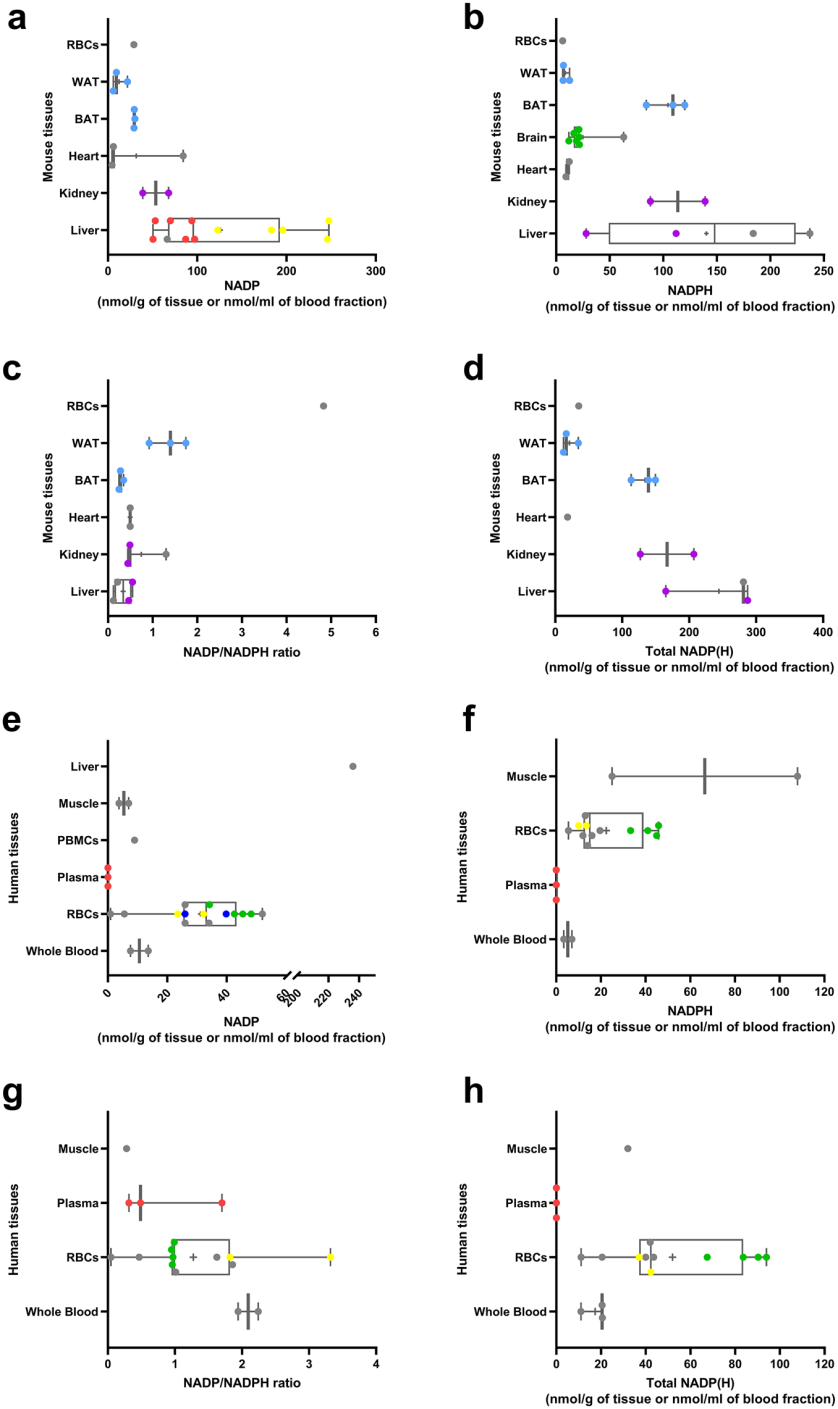
Reported mean physiological NADP^+^, NADPH, total NADP(H) and NADP^+^/NADPH ratio in normal mouse and human tissues. a-d: Reported mean physiological (**a**) NADP^+^, (**b**) NADPH, (**c**) NADP^+^/NADPH levels and (**d**) total NADP(H) in mouse tissues (nmol/g of tissue weight or nmol/ml of blood component) collected from young (< 14 months old) control mice. e–h: Reported mean physiological (**e**) NADP^+^, (**f**) NADPH, (**g**) NADP^+^/NADPH levels and (**h**) total NADP(H) in human tissues. The boxes represent the 25th to 75th percentiles with the median represented by the line inside the box. The means are shown as “ + ” symbol. The whiskers cover the minimum to maximum values. For each study that included more than one measurement per tissue, similar colors were assigned to the corresponding datapoints. Datapoints colored in grey represent studies with just one reported measurement for a given tissue. *PBMCs* peripheral blood mononuclear cells, *RBCs* red blood cells, *WAT* white adipose tissue, *BAT* brown adipose tissue.

The physiological NADP(H) levels in rodent tissues were compiled, with the most common measurement being in liver. Mouse liver exhibited a median value of 124.2 nmol/g of NADP^+^ (n = 13) and 140.2 nmol/g of NADPH (n = 4) (Fig. 4a,b), while rat liver showed a median of 95.11 nmol/g of NADP^+^ (n = 10) and 240.7 nmol/g of NADPH (n = 9) (Supplementary Fig. 5a,b). The median NADP^+^/NADPH ratio, derived from studies that measured both metabolites, was lower than 1 in most mouse and rat tissues (Fig. 4c, Supplementary Fig. 5c). The median total NADP(H) (NADP^+^ + NADPH) levels in mouse and rat liver were 244.4 (n = 3) (Fig. 4d) and 342.1 (n = 9) (Supplementary Fig. 5d), respectively. In humans, the most common measures of NADP(H) were performed in red blood cells showing a median of 33.2 nmol/ml for NADP^+^ (n = 14) and 22.4 nmol/ml for NADPH (n = 12) from non-matching studies, with a median NADP^+^/NADPH ratio of 1.27 and a median total NADP(H) of 52.0 nmol/ml from studies that measured both metabolites (n = 11) (Fig. 4e–h). Overall, the amount of data available for NADP(H) is sparce in all species leading to lower confidence in the median values for each tissue.

### Analysis of bias and variability in NAD(P)(H) quantification methods

**Figure 5:**
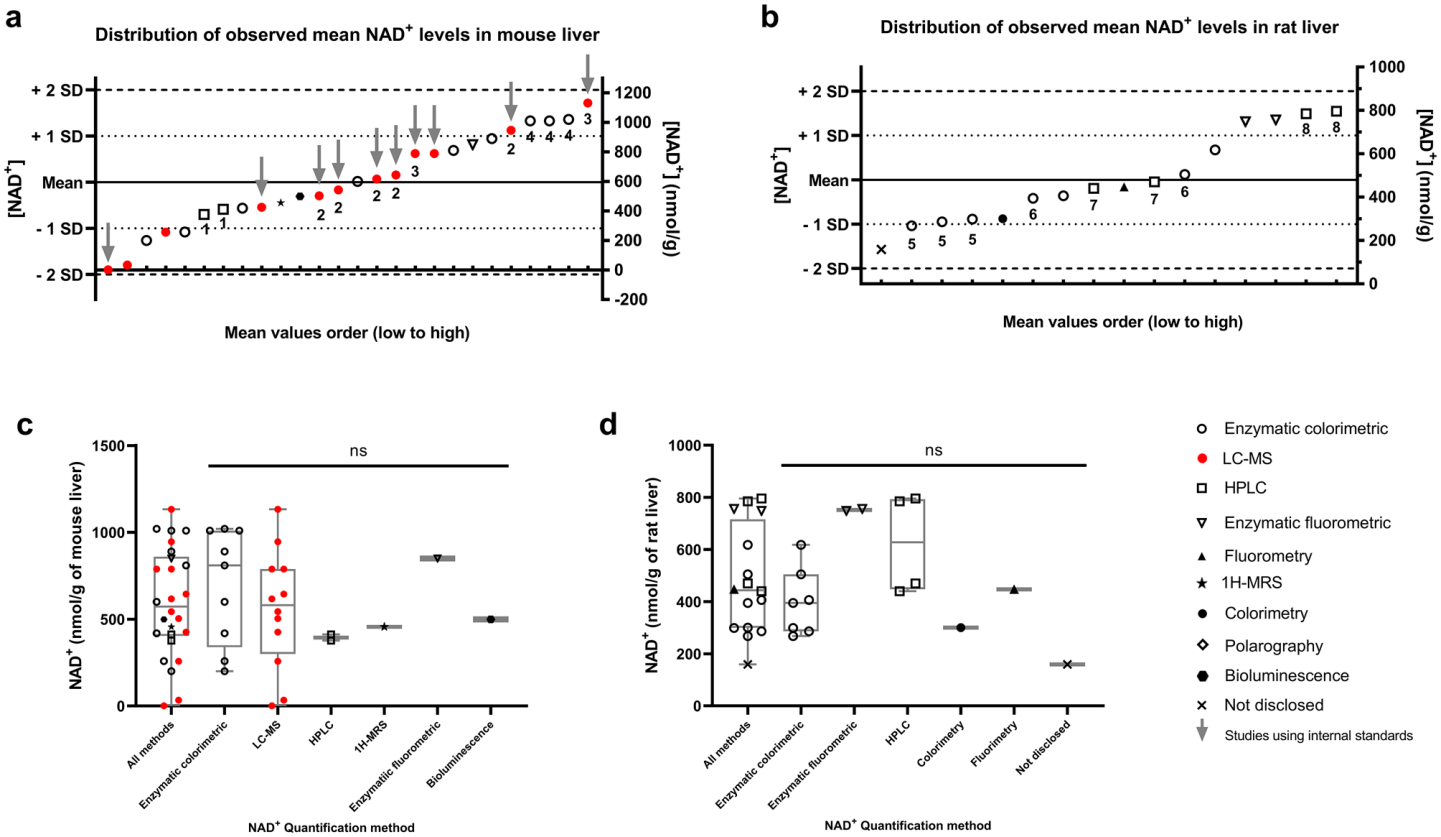
Distribution histogram of reported physiological NAD^+^ levels in normal mouse and rat liver. Mean NAD^+^ values measured using different quantification methods in (**a**) mice and (**b**) rat liver sorted from lowest to highest value (nmol/g of tissue weight). The represented data was collected from young control mice (< 14 months old) and rats (< 18 months old). For each study that included more than one measurement per tissue, the datapoints are labeled using a number that represent the study, respectively: 1: Gaikwad et al. (2001), 2: Dall et al. (2019), 3: Trammell et al. (2016), 4: Dietrich et al. (1968), 5: Ballard (1971), 6: Slater, Sawyer & Sträuli (1964), 7: Wendt et al. (2019), 8: Kiehlbauch et al. (1993). Arrows indicate LC–MS measurements that included the use of internal controls. NAD^+^ levels in (**c**) mouse and (**d**) rat liver segregated by quantification method. A One-way ANOVA with Sidak post hoc test was conducted to compare effect of the quantification methods on NAD^+^ levels and did not show any significant differences between methods.

Given that a majority of the studies included in this meta-analysis examined NAD^+^ levels in rodent liver samples (Fig. 1c,e), we used both mouse and rat liver physiological NAD^+^ levels to identify any potential bias in the results that corresponds to the quantification method (Fig. 5a,b). The mean age of mice included in this analysis were 17.9 weeks (Range: 2–55 weeks), with 6 studies not disclosing the age of the animals. For rats, most studies did not disclose the age of the animals except three that ranged from 2 days to 8 months. None of the studies reported the use of old animals. In mouse liver, the large range of the reported concentrations for NAD^+^ spanned from 1.8 to 1132 nmol/g of liver with a mean value of 596.0 (n = 26) (Fig. 5a), while NADH concentrations ranged from 0.9 to 109 nmol/g of tissue with a mean value of 66.43 (n = 6) (Supplementary Fig. 6a). Importantly, a coefficient of variation of 52.5% and 76.5% was calculated for the mean physiological NAD^+^ and NADH results in mouse liver, respectively, as measured by multiple quantification methods, showing the extent of the variability across studies. As predicted from these results, the mean NAD^+^/NADH ratio in mouse liver (3.41 + /− 3.16 S.D., n = 19) demonstrated an equally large range of variability with a C.V. of 92.7% (Supplementary Fig. 6c). Similar variability in NAD^+^ and NADH measures were also observed in rat liver (CV = 42.6% for NAD^+^ and CV = 44.4% for NADH). The corresponding concentrations for NAD^+^ and NADH in rat liver ranged from 159 to 796 (n = 16) and 65 to 265 (n = 12) nmol/g, respectively (Fig. 5b, Supplementary Fig. 6b), while the NAD^+^/NADH ratios exhibited a C.V. of 50.1% (Mean: 3.219 + /− 1.612 S.D., n = 23) (Supplementary Fig. 6d). The mean values and standard deviations for NAD^+^, NADH, total NAD(H) and the NAD^+^/NADH ratio data in mouse and rat liver, grouped by quantification method, are shown in Supplementary Table 2. Concerning NADP(H) levels and the NADP^+^/NADPH ratio, similar variability was observed in liver tissues. NADP^+^ levels ranged from 50.4 to 247.4 nmol/g of tissue for mice (Supplementary Fig. 8a) and from 45.7 to 171 nmol/g of tissue for rats (Supplementary Fig. 8b), while NADPH levels ranged from 28 to 236.9 nmol/g of tissue for mice (Supplementary Fig. 8c) and from 90 to 409 nmol/g of tissue for rats (Supplementary Fig. 8d). Lastly, the NADP^+^/NADPH ratio ranged from 0.12 to 0.55 in mouse liver (Supplementary Fig. 8e) and from 0.12 to 1.44 in rat liver (Supplementary Fig. 8f).

When specifically examining the variability of NAD^+^ measures there was no significant differences between any particular quantification method in mice or in rats (Fig. 5c,d). Even when considering variability of the mouse liver NAD^+^ data from LC–MS measurements, the method with the best array of potential controls, culminated in the largest range of results (1.8 to 1132.3 nmol/g). Even the most recent LC–MS studies examined exemplified the range of these NAD^+^ values, including values of 1.8 nmol/g^41^ and 33.8 nmol/g^42^ and much higher values of 946.3 nmol/g^43^ and 1132.3 nmol/g^44^. In addition, the large range of LC–MS NAD^+^ values did not appear to correlate to studies that excluded isotope labeled internal standards (Fig. 5a), a method used to improve the identification of the metabolite and accuracy of the quantified value. However, the two identified LC–MS studies that relied solely on an external standard curve for NAD^+^ quantification, rather than including an internal control, were on average lower than the mean for all measures (Fig. 5a). Further, the studies using internal standard normalization used different types of internal standards, such as the inclusion of a single labeled reference metabolite^45,46^ or labeled metabolite extracts from yeast^43,44^ or cell cultures^41^. Interestingly, these studies that used yeast or cell culture extract derived standards provided either the highest or lowest LC–MS derived measures for liver NAD^+^ levels. This potentially emphasizes how cellular extracts of isotope rich standards can contribute to the variability in NAD^+^ measurements as the matrix effect of the added standard yeast or cellular extract could interfere with the ionization efficiency and consequently the measured concentrations of the target metabolites.

The limitations for this analysis of quantification bias and variability include potential differences in environment, genetic background, sacrifice, sex, and age. All data for this analysis came from animals on chow diet but may differ in type. Upon sacrifice there are reported differences in fed or fasted state of the animal and time of day for sacrifice. Additionally, although most mouse studies were performed on a C57BL/6 background, there was a larger range of backgrounds used for rats across studies (Wistar: 38%, Albino Wistar: 25%, and 6% for Hooded Wistar, Sprague–Dawley, Holtzman, ACI, Long-Evans and Zucker rats). Across mouse liver studies, there were no statistical differences between male or female values for NAD^+^, NADH or total NAD(H) levels or for the NAD^+^/NADH ratio (Supplementary Fig. 7). However, we cannot draw conclusions due to the limited number of female cohorts.

### The effect of pre-mortem versus post-mortem tissue collection on NAD(P)(H) levels

**Figure 6:**
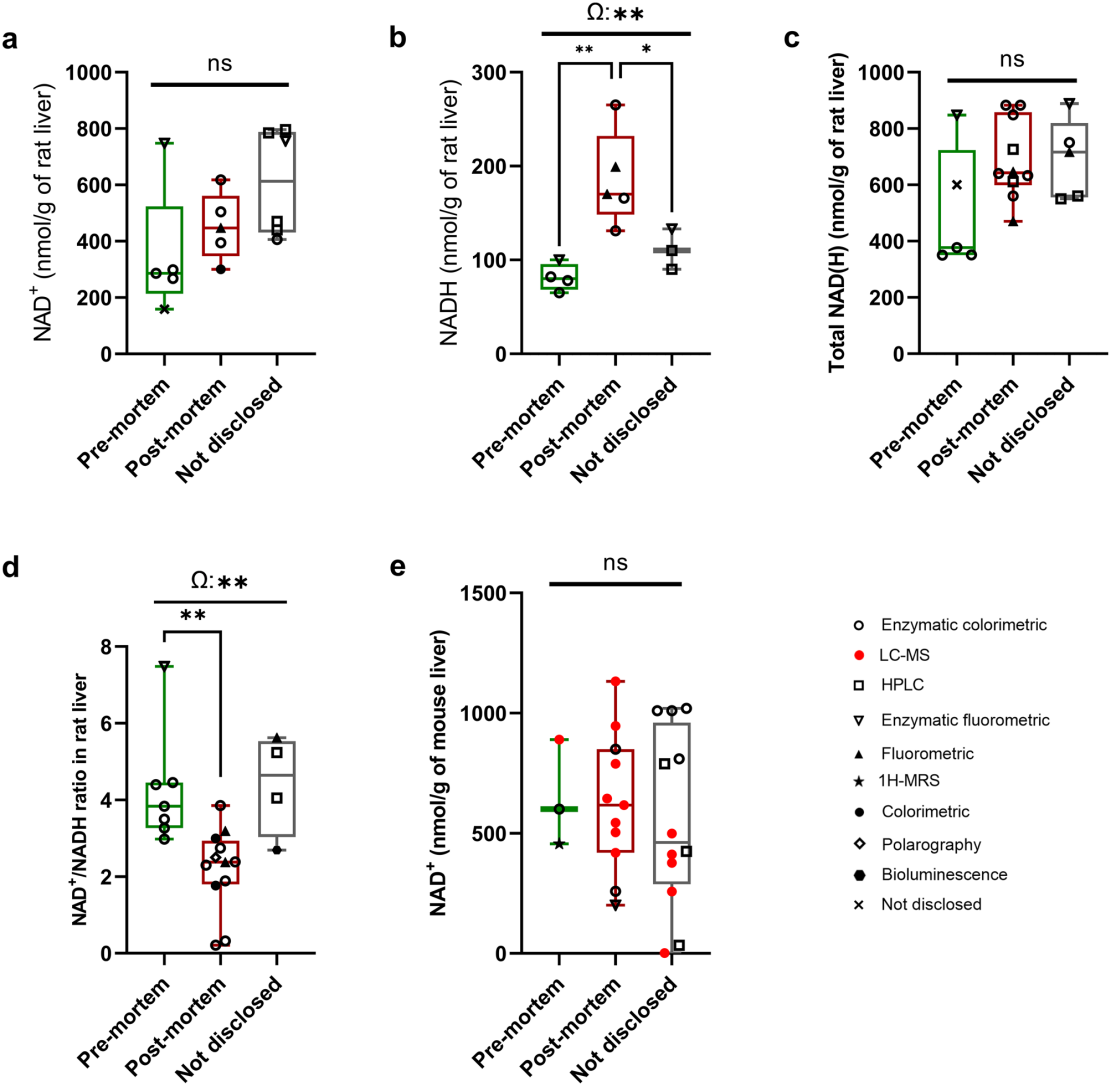
Effect of pre- versus post-mortem tissue collection on NAD(H) levels in normal rats and mice liver. a-d: Reported (**a**) NAD^+^, (**b**) NADH, (**c**) total NAD(H) levels, and (**d**) NAD^+^/NADH ratio in young (< 18 months old) control rat liver samples harvested at different timepoints relative to sacrifice. (**e**): NAD^+^ levels in young (< 14 months old) control mouse liver samples harvested at different timepoints relative to sacrifice. Boxes represent 25th and 75th percentile with median line. Whiskers show min. to max. values. Statistical significance was determined by one-way ANOVA with multiple comparisons using Tukey’s test as post hoc test, setting P < 0.05 (*) and P < 0.005 (**) as significant. Ω: Overall effect of harvest timepoint relative to sacrifice.

We predicted that there may be some observable difference in NAD(P)(H) redox state that is dependent on tissue harvesting procedures. To answer this question, we examined the effect of pre- versus post-mortem tissue collection procedures on NAD(P)(H) redox status. For this analysis we compared values from rat liver tissues given the larger number of studies defining their sacrifice protocol. When disclosed, all the reviewed studies stated that the tissue samples were kept cold and immediately extracted on ice or frozen at − 80 °C prior to NAD(P)(H) measurements. This analysis determined that there was no significant difference in NAD^+^ concentrations in rat liver (Fig. 6a) between tissues extracted before or after euthanasia. However, the reported NADH levels in rat liver are significantly higher in post-mortem samples (Fig. 6b). No differences in total NAD(H) levels (Fig. 6c) were observed but the NAD^+^/NADH ratio was lower in tissues collected following euthanasia (Fig. 6d), which is consistent with the variation observed in NADH levels. NADP^+^, NADPH, and the NADP^+^/NADPH ratio results in rat liver grouped by tissue harvest timepoint are shown in Supplementary Fig. 9; however, the insufficient number of pre-mortem samples do not allow us to interpret the effect of tissue harvest timepoint on NADP(H) levels. In mouse liver, there was no difference between pre- and post-mortem NAD^+^ levels, however the number of reporting studies were limiting (Fig. 6e). No NADH, NADP^+^, or NADPH pre-mortem data in mouse liver was available for comparison.

## Discussion

Many recent studies have demonstrated that NAD(P)(H) regulation is essential for cellular homeostasis and redox status. Data obtained from rodents have led to studies examining human blood NAD^+^ levels, as a less invasive indicator of whole-body NAD^+^ levels. Recent clinical studies demonstrated that NAD^+^ levels were reduced in disease states^23,47^ and elevated following NAD^+^ boosting strategies^23,44,48,49^. We performed a meta-analysis of all studies published between 1946 and June 20, 2021 that contained quantitative data for NAD(P)(H) levels in mammalian species with a special focus on mice, rats, and humans as they represent the most studied species in the biomedical field. This analysis was done in part to determine the mean standard level of NAD(P)(H) concentrations in normal mammalian tissues given the well-known variance in these measures across studies. We hope that these data can be used to stimulate standardized protocols in the field of NAD^+^ research and to promote the use of NAD(P)(H) levels and redox ratios as reliable biomarkers for disease and treatment regimens.

Despite considerable variability in measures across rodent tissues this meta-analysis displayed similar mean NAD^+^ levels across tissues with some exceptions. Interestingly, mouse skeletal muscle exhibited lower median NAD^+^ levels (Fig. 2a) than other highly metabolic tissues (i.e., liver, kidney, heart, and brain). This may indicate different NAD(H) redox status in skeletal muscle or may point to greater issues with metabolite extraction in fibrous tissues. However, these observations could not be verified in humans due to a lack of sampled tissues beyond blood and muscle.

There are many potential factors that could affect the accuracy of physiological NAD(P)(H) measurements in tissue samples. When reported, all studies described tissues as being harvested and directly submerged into liquid nitrogen, or kept on ice before freezing or processing in order to preserve the heat-sensitive fractions (NAD^+^ and NADP^+^). The effect of pH on the various NAD(P)(H) fractions, such as the acid-labile nature of the reduced NAD(P)H forms, has lead to the use of pH-neutral extraction solvents by the majority of the studies. However, given the rapid interconversion of the reduced and oxidized metabolites and the effect of anoxia on NAD(H)^50–53^, it is also important to ensure rapid extraction of tissue metabolites and the appropriate procedures for quenching cellular NAD(P)(H)-redox/consumption enzymes, such as through deproteinization. Along these lines, our analysis of pre- versus post-mortem tissue collection indicates that post-mortem analysis favors the reduction of NAD^+^. The reduction of tissue NAD^+^ has been previously described in vivo in the brain, heart and liver of rats exposed to an extended 2.5-h hypoxic atmosphere^54^, an environment that may be partially recapitulated by extended periods of post-sacrifice tissue collection before snap freezing. This may indicate that extracting tissues while under anesthetic, or prioritizing the immediate harvest of tissues intended to be used for metabolite analysis post-sacrifice, should occur to obtain representative NAD(P)(H) measurements.

There is also the potential for various quantification methods, each having different limitations, to contribute to the overall intra-study variability of NAD(P)(H) measurements. In the last two decades the most frequently used methods were enzyme cycling assays, LC–MS, and HPLC. Each of these methods are affected by metabolite extraction techniques, quantification parameters, and the implementation of proper quality controls. Using LC–MS, Lu *et al*. have shown that the interconversion between reduced and oxidized forms of NAD(P)(H) metabolites occurs at different rates in various extraction buffers or solvents with the acetonitrile:methanol:water with 0.1 M formic acid mixture yielding the highest recovery with the least interconversions^31^. However, when using LC–MS this interconversion can be monitored between individual samples of a study by spiking with internal controls such as NAD(P)(H) isotopes, which is an advantage of the LC–MS technique over that of HPLC and enzyme cycling assays^31,33,55^. Nonetheless, even with well controlled LC–MS techniques, various factors can interfere with the measured signal, including matrix effects and variations in ionization efficiency. For example, some studies use ^13^C -labeled yeast extracts as internal standards in LC–MS based metabolomics. However, spiking the extracted sample metabolite matrix with a ^13^C-labeled yeast extract metabolite matrix can have various consequences, such as ion suppression, which reduces the signal of labeled and/or unlabeled metabolites, and can lead to errors in the absolute quantification if not detected by a thorough quality assessment^56^. Also, although LC–MS techniques theoretically allow for the measurement of multiple NAD(P)(H) metabolites in one experiment, there are still limitations since optimal dilutions must be run if the metabolites of interest have large differences in concentrations. Thus, despite their advantages over the simpler enzyme cycling assays, the complexity of LC–MS methods imposes the necessity for thorough optimization. Recently new analytical methods, such as, NAD^+^ biosensors and imaging-based mass spectrometry, have been developed but there is still insufficient quantitative data generated from these techniques to include in a meta-analysis. Although, one study included in our meta-analysis used a paper-based bioluminescent biosensor to measure NAD^+^ levels in mouse liver and other sample types^57^. This study was included in the bioluminescent assays group due to the similar method of detection (Fig. 1a).

For the purpose of this analysis, data from 677 studies were excluded. This included studies that provided relative NAD(P)(H) results (51%) rather than quantitative, as well as studies in which NAD(P)(H) concentrations were not normalized to tissue weight, protein content, or blood volume (13%). 36% of the excluded studies were performed on non-suitable specimens (e.g., non-mammalian subjects, cell or tissue cultures). Beyond the excluded studies, major limitations in this meta-analysis include the variation of, or lack of reported information related to, pre-analytical procedures (pre- or post-mortem extraction or extraction buffer information) and/or the analytical methods, along with the age, sex, genetic background, diet and feeding status at sacrifice for rodents.

Although our meta-analysis highlights the inter-study variability and the necessity to standardize NAD(P)(H) quantitative measurements, our analysis does not assess or debate the validity of the comparative results within an individual study. Given the implications of multiple NAD(P)(H) metabolites serving as biomarkers to health in humans, the standardization or thorough study optimization of pre-analytical and analytical procedures will allow for better comparisons of results across studies. This becomes even more crucial with an increasing number of clinical studies testing the effect of drugs or supplements used to alter tissue NAD(P)(H) levels to improve health in humans.

## Methods

### Literature search

“Epub Ahead of Print”, “In-Process & Other Non-Indexed Citations”, “Versions”, “PubMed-Not-MEDLINE”, “Daily update”, “Front segment weekly update”, “Back files from 1946 to start of front segment” citations between 1946 and June 20, 2021 in the MEDLINE ALL (Ovid) database were searched and reviewed for articles containing quantitative data of NAD(P)(H) metabolite levels in mammalian tissues as outlined in Appendix S1. The search query strategy yielded 3377 articles. An additional blood focused search was performed using the same database and yielded 1513 articles (Appendix S2).

### Screening methodology

Abstracts and titles reporting NAD(P)(H) metabolite quantification in mammalian tissues and blood underwent full-text screening. All abstract reviews were verified with a second review and any conflicts resolved. Following abstracts screening 643 publications were selected for full-text review for the tissue-focused search and 272 for the blood-focused search (Supplementary Fig. 10). Studies that only presented relative NAD(P)(H) metabolite data were excluded during the full-text screening, with the exception of studies reporting NAD^+^/NADH ratios. Results derived strictly from tissues, cells, isolated organelles maintained in media, or from blood effluent from perfusion were excluded. This full-text review led to the exclusion of 667 (457 + 210) articles due primarily to the lack of NAD metabolite data (50.5%), data derived from non-suitable specimens (36.3%), or data presented as relative/arbitrary units (13.2%). Finally, 241 articles were included in the qualitative meta-analysis and 205 in the quantitative meta-analysis (Supplementary Fig. 10). Although our initial search included studies published from 1946 to June 20, 2021. As mentioned above, the oldest study with quantitative NAD(P)(H) data that met our acceptance criteria was published in 1961.

### Data extraction

Numerical data for NAD(P)(H) metabolites were either obtained directly from the articles or extracted from article figures using a semi-automatic data extraction software (WebPlotDigitizer^58^). Additionally, where applicable, the tissue sampling timepoints relative to sacrifice and/or tissue/blood sampling methods (i.e. method of anesthesia and/or euthanasia, tissue handling, and storage temperature) were recorded. For animal models, the species, characteristics (e.g. strain, genotype, age, and weight), and environmental conditions (e.g. sleep cycle, type of diet, feeding frequency, and feeding state relative to tissue sampling) were recorded. Additionally, for all studies, treatments (e.g. pharmacological treatments, surgeries, irradiation, tumor induction and other procedures applied to the study subjects) and all study group information (e.g. treatment or disease groups and corresponding controls) were extracted. Finally, the method of NAD(P)(H) quantification method (e.g. enzymatic assays, mass spectrometry based, HPLC, NMR, bioluminescence) and publication specifics (i.e. publication title, reference code [DOI, Medline UI, PMID] or hyperlink and year of publication) were noted (Supplementary Material 2).

### Data analysis

Prior to analysis, all concentrations were converted to nmol/g of tissue or nmol/g of proteins for tissue samples or nmol/ml for blood fractions. Due to the low number of results normalized to protein content, we only show results normalized to tissue weight and blood volume. Statistical analysis was performed with GraphPad Prism version 9.3.1 (GraphPad Software Inc., San Diego, California, USA, www.graphpad.com).

## Supplementary Information

Supplementary Information 1.

Supplementary Information 2.

## Data Availability

All data extracted and analysed during this study are included in this published article and its supplementary information files.
