# Supplementary material for: Meta-analysis of NAD(P)(H) quantification results exhibits variability across mammalian tissues
Source: Sci Rep. 2023 Feb 11;13:2464. doi: 10.1038/s41598-023-29607-8 (PMC9922293; doi:10.1038/s41598-023-29607-8)
Supplement: Supplementary file 1 — Supplementary Information 1. [file 41598_2023_29607_MOESM1_ESM.docx]

| Human samples | **NAD^+^**  (nmol/g of tissue or nmol/ml of blood) | **NADH**  (nmol/g of tissue or nmol/ml of blood) | **Total NAD(H)**  (nmol/g of tissue or nmol/ml of blood) | **NAD^+^/NADH ratio** |
| --- | --- | --- | --- | --- |
| Whole blood | 44.62 +/- 18.69 (n=23) | 13.49 +/- 15.29 (n=2) | 28.94 +/- 16.16 (n=6) | 9.066 +/- 9.405 (n=3) |
| Plasma | 0.3718 +/- 0.392 (n=8) | 0.387 +/- 0.429 (n=8) | 60.23 +/- 178.1 (n=9) | 1.570 +/- 1.031 (n=7) |
| RBCs | 46.96 +/- 9.947 (n=7) | 1.75 +/- 0.957 (n=7) | 49.66 +/- 9.88 (n=9) | 23.65 +/- 12.94 (n=8) |
| PRBCs | 79.80 +/- 34.14 (n=3) | 44.4 (n=1) | 127.8 nmol/ml PRBCs (n=1), 3149 nmol/mmol PRBCs hemoglobin) | 0.53 (n=1) |
| PBMCs | 12.16 (n=1) | / |  | / |
| Serum | / | / | 2.327 (n=1) | / |
| Skeletal Muscle (Wet) | 191.9 +/- 18.62 (n=4) | 121.8 +/- 65.51 (n=4) | 620.8 +/- 388.5 (n=4) | 6.137 (n=1) |
| Skeletal Muscle (Freeze-Dried) | 1713 +/- 150.4 (n=3) | 136.8 +/- 42.89  (n=6) | 1853 +/- 180.2 (n=3) | 12.7 +/- 2.338 (n=3) |
| Placenta (term) | / | / | 591 (n=1) | 3.92 (n=1) |
| WAT | 7.15 (n=1) |  | / | / |
| Breast tissues | / | 440 (n=1) | / | / |
| Oral cavity biopsy | / | 820 (n=1) | / | / |
| Liver (from living healthy donors) | / | / | / | 0.775 (n=1) |

**Supplementary table 1: Mean physiological NAD(H) levels measured in human samples.** All results are expressed as mean +/- S.D. n = number of control groups.


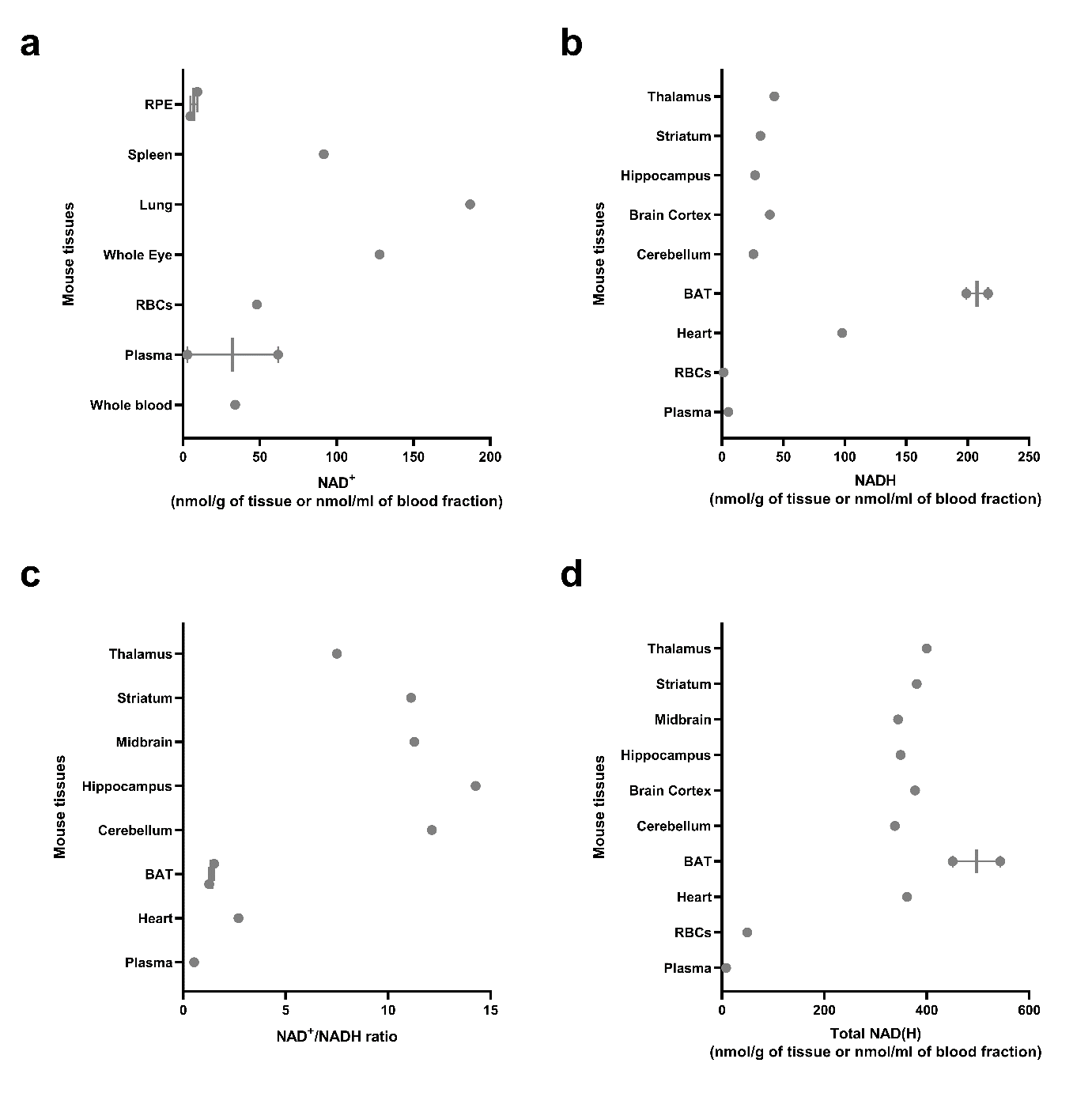


**Supplementary** **figure 1:** **Reported physiological NAD^+^, NADH, total NAD(H) and NAD^+^/NADH ratio in mouse tissues with n<3.** a-d: Reported mean (a) NAD^+^, (b) NADH, (c) NAD^+^/NADH levels and (d) total NAD(H) in various mouse tissues collected from young control mice (<14 months old). BAT: Brown adipose tissue, RBCs: Red blood cells, RPE: Retinal pigmented epithelium.


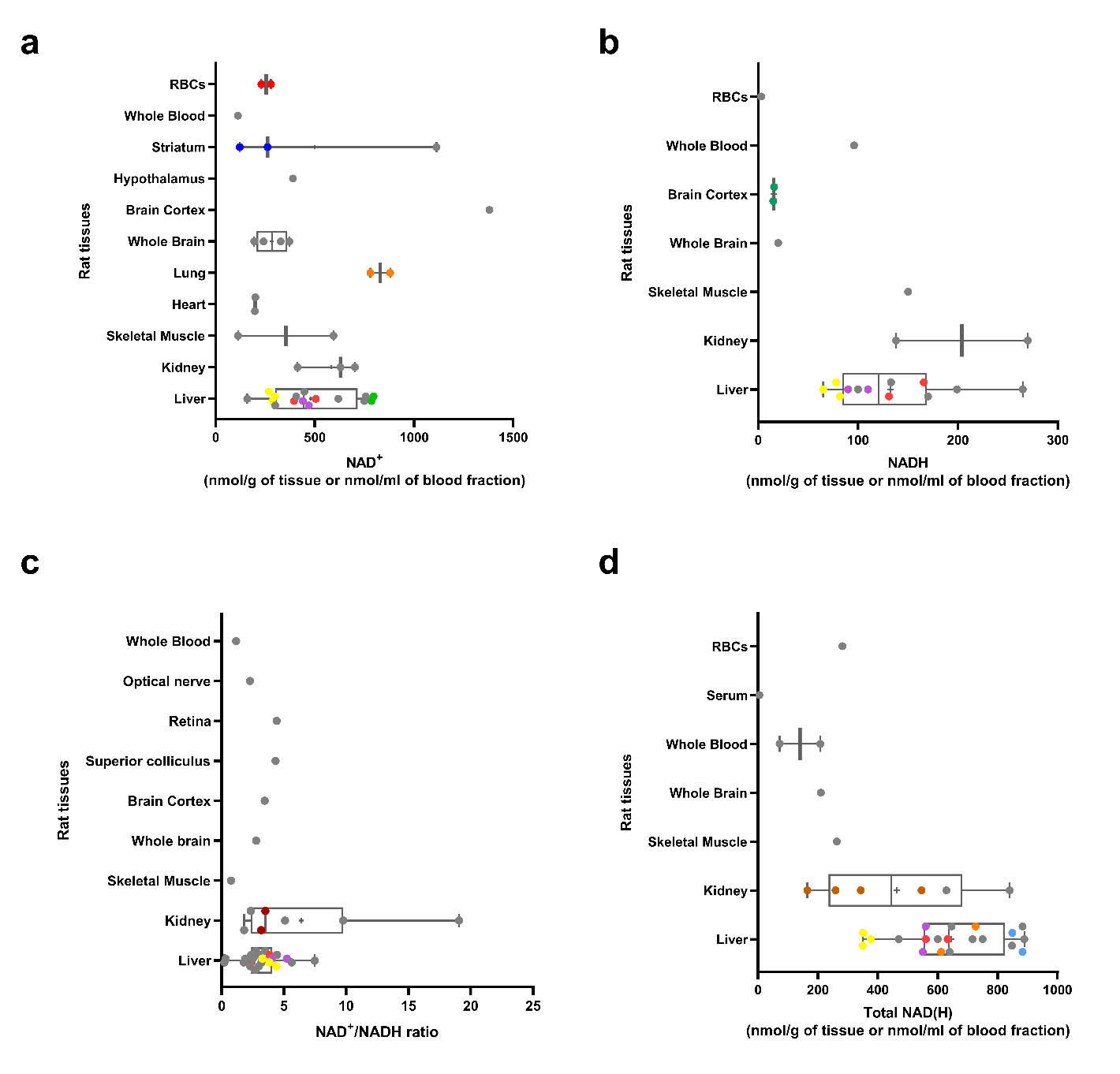


**Supplementary figure 2: Observed physiological NAD^+^, NADH, total NAD(H) and NAD^+^/NADH ratio in rat tissues.** Reported mean physiological (a) NAD^+^ and (b) NADH levels, (c) NAD/NADH ratios and (d) total NAD(H) in various rat tissues collected from young control rats (<18 months old). The boxes represent the 25^th^ to 75^th^ percentiles with the median represented by the line inside the box. The mean values are shown as “+”. The whiskers cover the minimum to maximum values. For each study including more than one control group, similar colors were assigned to the corresponding datapoints. RBCs: Red blood cells.

**
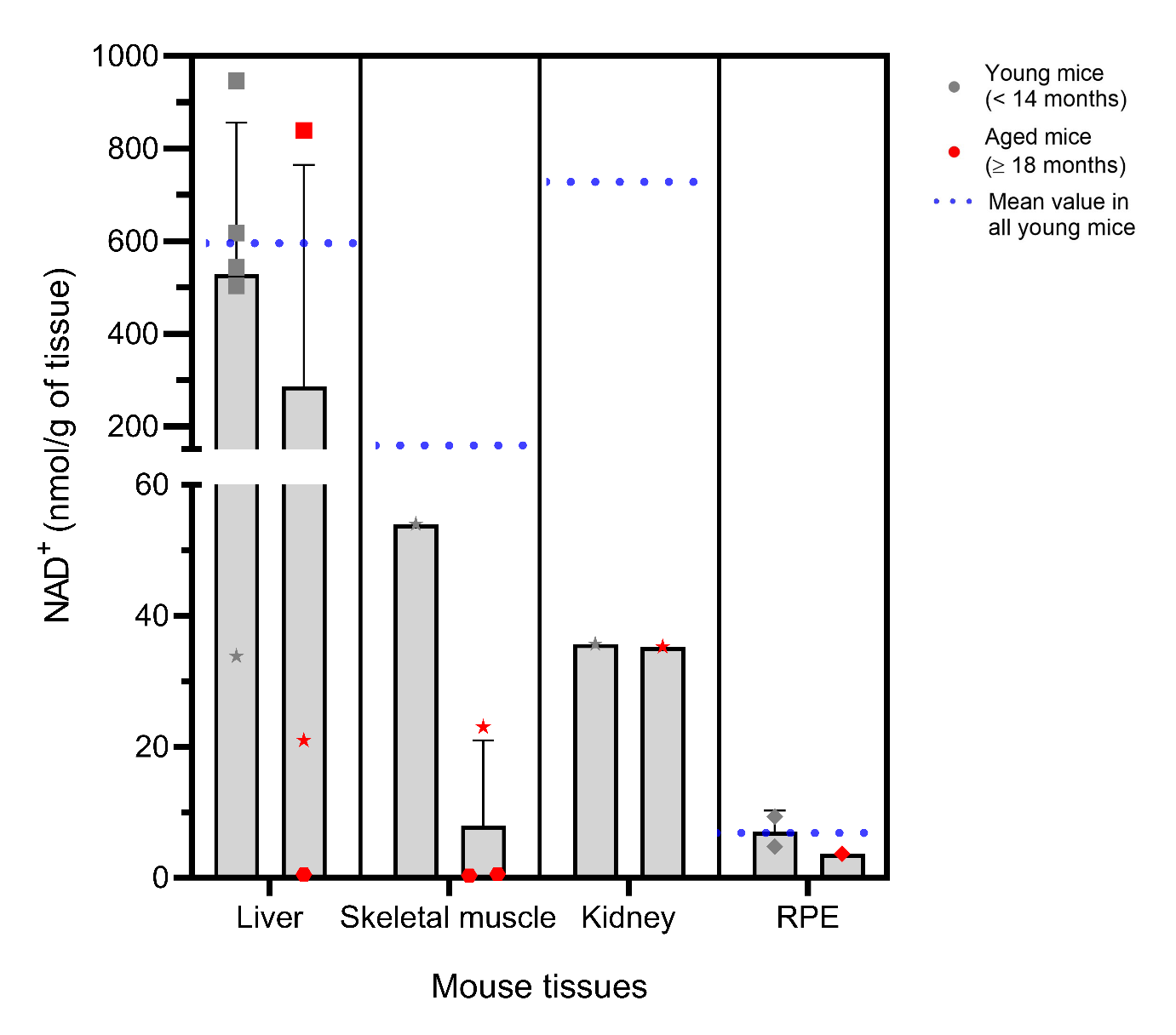
**

**­­­­**

**Supplementary figure 3:** Mean physiological NAD^+^ levels in young mice (<14 months) compared to the corresponding aged mice (18 months or older) from the same studies. Each study is represented by a specific datapoint shape. The mean physiological NAD^+^, NADH, total NAD(H), and the NAD^+^/NADH ratio in all young mice data is represented by the dotted lines.

**
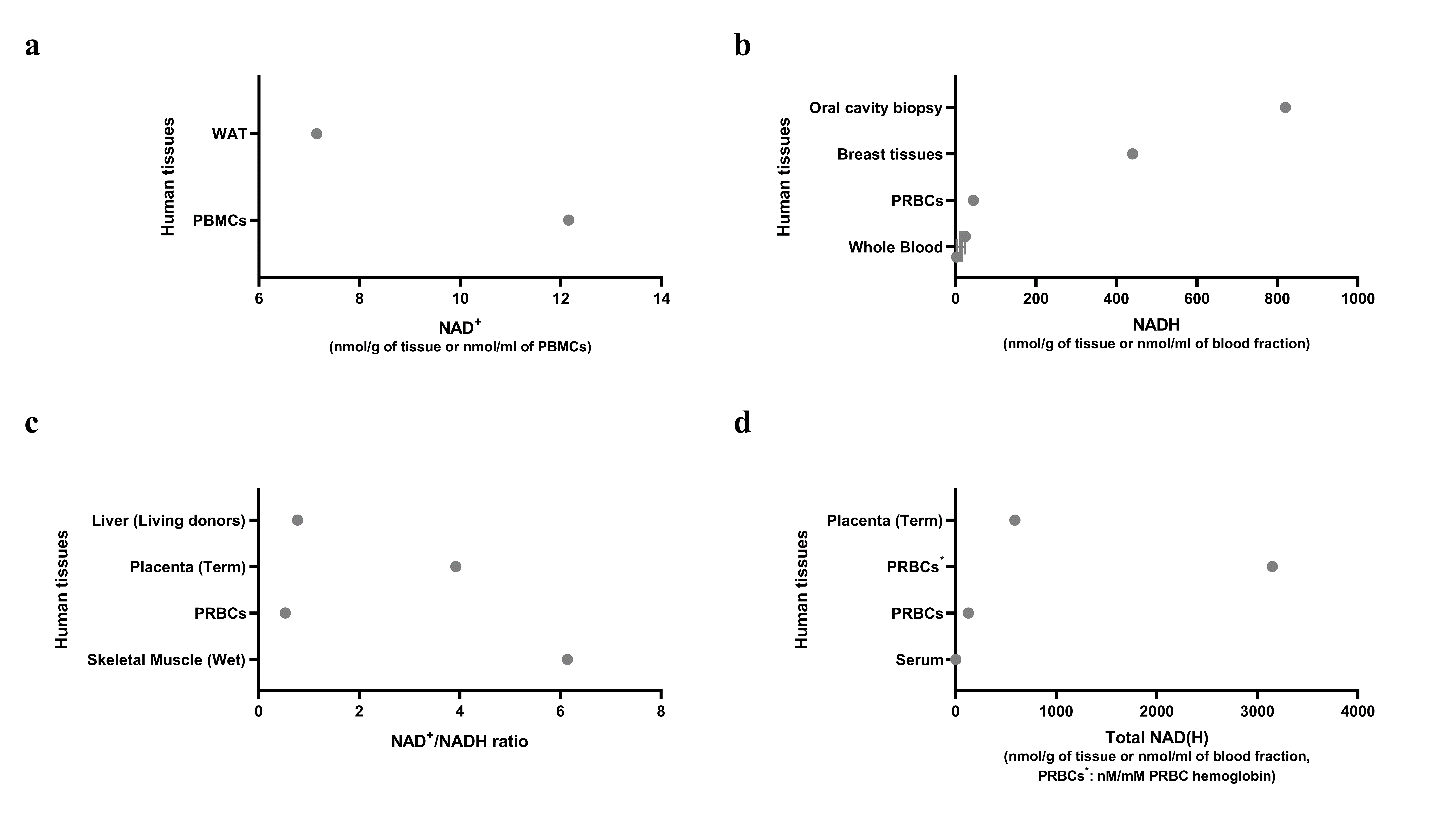
**

**Supplementary figure 4: Reported physiological NAD^+^, NADH, NAD^+^/NADH ratio, and total NAD(H) in human tissues with n<3.** a-d: Reported mean (a) NAD^+^, (b) NADH, (c) NAD^+^/NADH and (d) total NAD(H) levels in various human tissues. PBMCs: Peripheral blood mononuclear cells, PRBCs: Packed red blood cells, RBCs: Red blood cells.

**
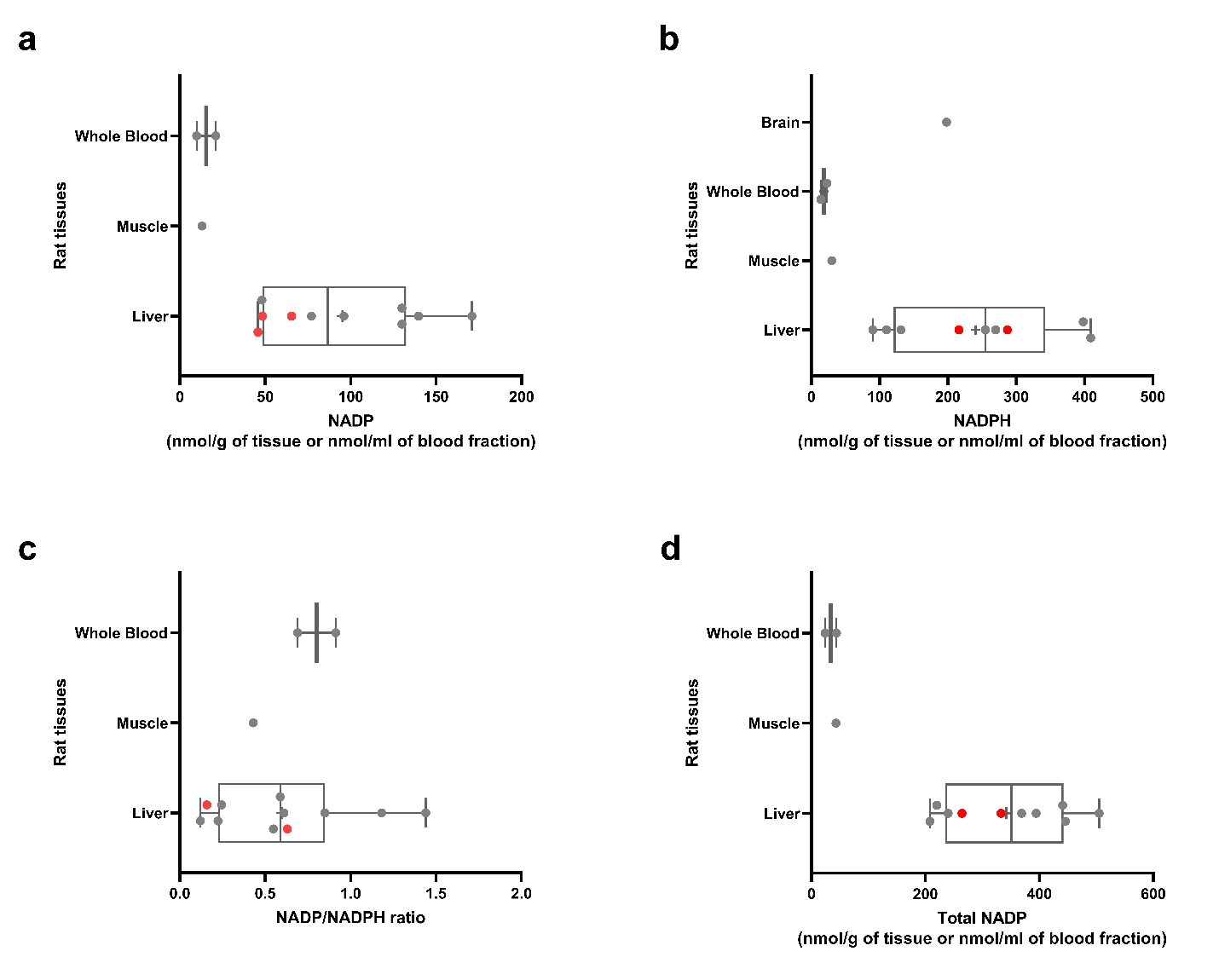
**

**Supplementary figure 5:** **Reported physiological (a) NADP and (b) NADPH levels, (c) NADP/NADPH ratio, and (d) total NADP(H) in various rat tissues.** Data represents results obtained from young control rats (<18 months old). The boxes represent the 25^th^ to 75^th^ percentiles with the median represented by the line inside the box. The whiskers cover the minimum to maximum values. For each study including more than one control group, similar colors were assigned to the corresponding datapoints.

**
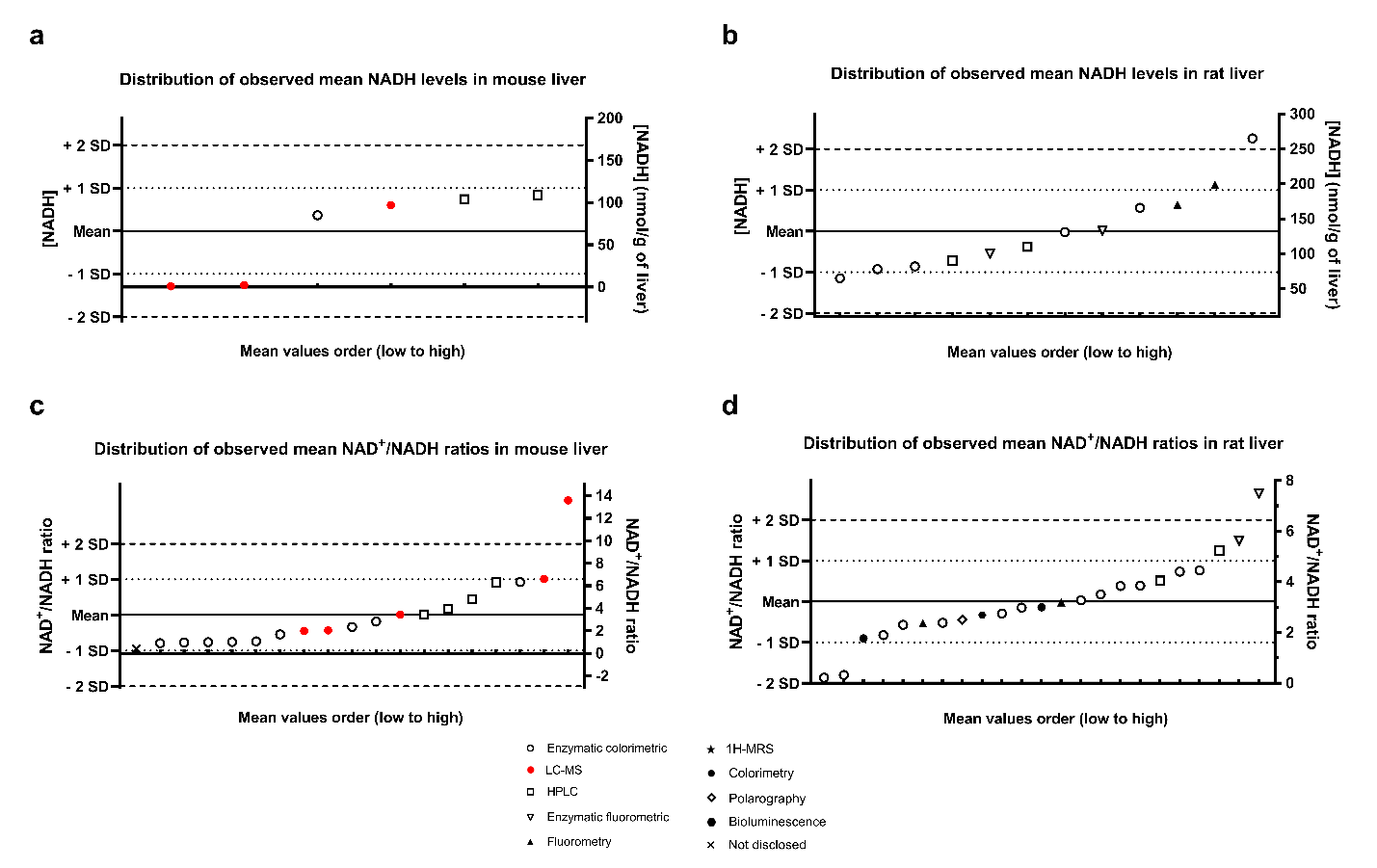
**

**Supplementary figure 6:** a,b: Distribution of mean NADH values measured using different quantification methods in (a) mouse liver with mean +/- S.D = 66.4 +/- 50.8 nmol/g of tissue (CV=76.5%) and (b) rat liver with mean +/- S.D = 132.4 +/- 58.7 nmol/g of tissue (CV=44.4%). c,d: Distribution of mean NAD^+^/NADH ratios measured using different quantification methods in (c) mouse liver with mean +/- S.D = 3.41 +/- 3.16 (CV=92.7%) and (d) rat liver with mean +/- S.D = 3.219 +/- 1.612 (CV=50.1%). Tissues were collected from young control animals: <14 months for mice and <18 months for rats.


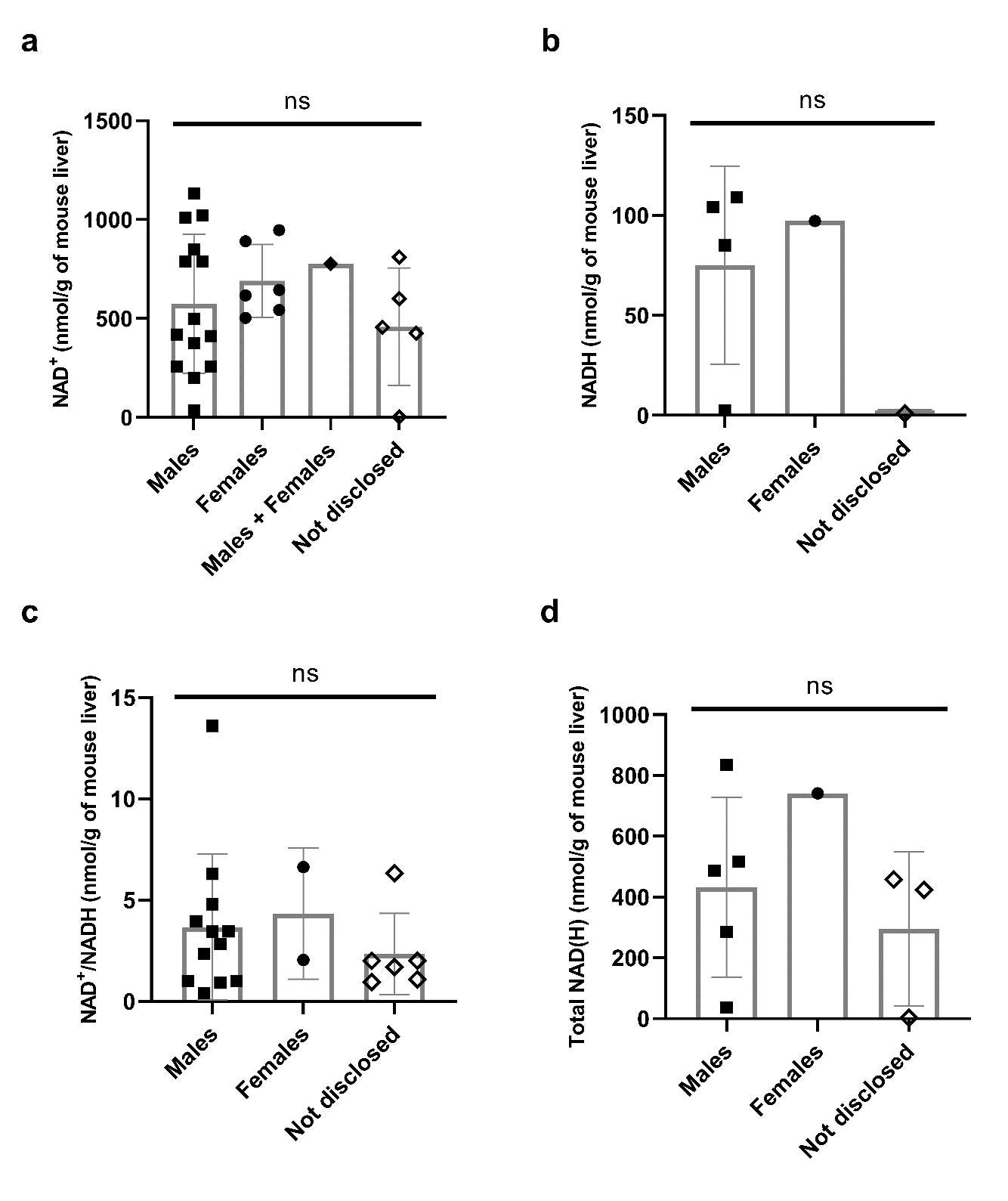


**Supplementary figure 7: Sex differences in NAD(H) metabolites levels in mouse liver. (a) NAD^+^, (b) NADH, (c) NAD^+^/NADH ratio, and (d) total NAD(H).** Results from mice older than 14 months were excluded. Statistical significance was determined by one-way ANOVA with multiple comparisons using Tukey’s test as post-hoc test. Error bars represent standard deviation.

**Supplementary table 2: NAD(H) levels (nmol/g of wet tissue weight) observed in control rats and mice liver samples.** All results are expressed as mean +/- S.D. n = number of studied groups. Results from old animals (Older than 14 months for mice and 18 months for rats) and from animals receiving high fat diets were excluded.

| **Species** | **Rat** | | | | **Mouse** | | | |
| --- | --- | --- | --- | --- | --- | --- | --- | --- |
| **Quantification method** | **NAD^+^** | **NADH** | **Total NAD(H)** | **NAD^+^/NADH ratio** | **NAD^+^** | **NADH** | **Total NAD(H)** | **NAD^+^/NADH ratio** |
| Enzymatic colorimetric | 396.7 +/- 128.5 (n=7) | 131.1 +/- 75.8 (n=6) | 627.7 +/- 214.9 (n=10) | 2.779 +/- 1.363 (n=13) | 690.9 +/- 330.8 (n=9) | 85.09 (n=1) | 389.1 +/- 91.33 (n=3) | 2.016 +/- 1.758 (n=9) |
| LC-MS | / | / | / | / | 556.9 +/- 342.9 (n=12) | 33.5 +/- 55.17 (n=3) | 389.3 +/- 427.5 (n=4) | 5.54 +/- 4.88 (n=5) |
| HPLC | 622.7 +/-194.1 (n=4) | 100.0 +/- 14.1 (n=2) | 612.0 +/- 80.8 (n=4) | 4.64 +/- 0.8 (n=2) | 394.5 +/- 24.75 (n=2) | 106.5 +/- 3.536 (n=2) | 612.3 +/- 193.4 (n=3) | 4.630 +/- 1.243 (n=4) |
| Enzymatic fluorimetric | 752.0 +/- 5.6 (n=2) | 116.5 +/- 23.3 (n=2) | 868.5 +/- 29.0 (n=2) | 6.55 +/- 1.3 (n=2) | 849.8 (n=1) | / | / | / |
| Colorimetry | 300.0 (n=1) | 170.0 (n=1) | 470.0 (n=1) | 2.38 +/- 0.86 (n=2) | / | / | / | / |
| Fluorimetry | 447.0 (n=1) | 199.0 (n=1) | 363.5 +/- 19.1 (n=2) | 2.78 +/- 0.58 (n=2) | / | / | / | / |
| Bioluminescence | / | / | / | 2.690 (n=1) | 499.3 (n=1) | / | / | / |
| Polarography | / | / | / | 2.500 (n=1) | / | / | / | / |
| 1H-MRS | / | / | / | / | 456.0 (n=1) | / | / | / |
| Not disclosed | 159.0 (n=1) | / | 600.0 (n=1) | / | / | / | / | 0.41 (n=1) |
| **All methods** | **479.9 +/- 204.4 (n=16)** | **132.4 +/- 58.7 (n=12)** | **644.7 +/- 174.9 (n=20)** | **3.219 +/- 1.612 (n=23)** | **596.0 +/- 312.6 (n=26)** | **66.43 +/- 50.82 (n=6)** | **456.1 +/- 287.6 (n=10)** | **3.41 +/- 3.16 (n=19)** |

**
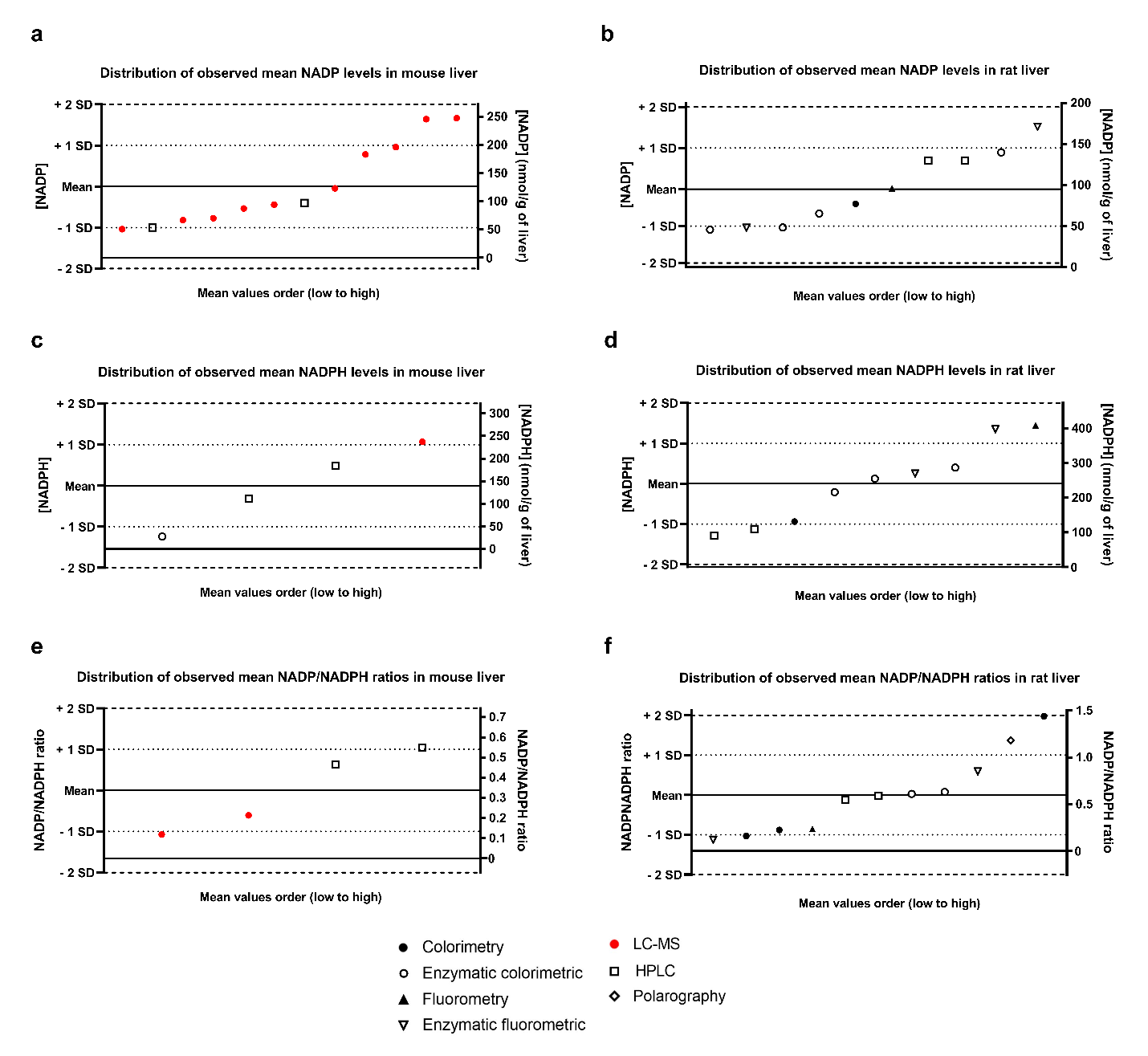
**

**Supplementary figure 8: Distribution histogram of reported physiological NADP(H) levels in mouse and rat liver.** Mean NADP (a,b), NADPH (c,d), and NADP/NADPH (e,f) values measured in mouse and rat liver, respectively, using different quantification methods. Values are sorted from lowest to highest value (nmol/g of tissue weight). Tissues were collected from young control animals: <14 months for mice and <18 months old for rats.

**
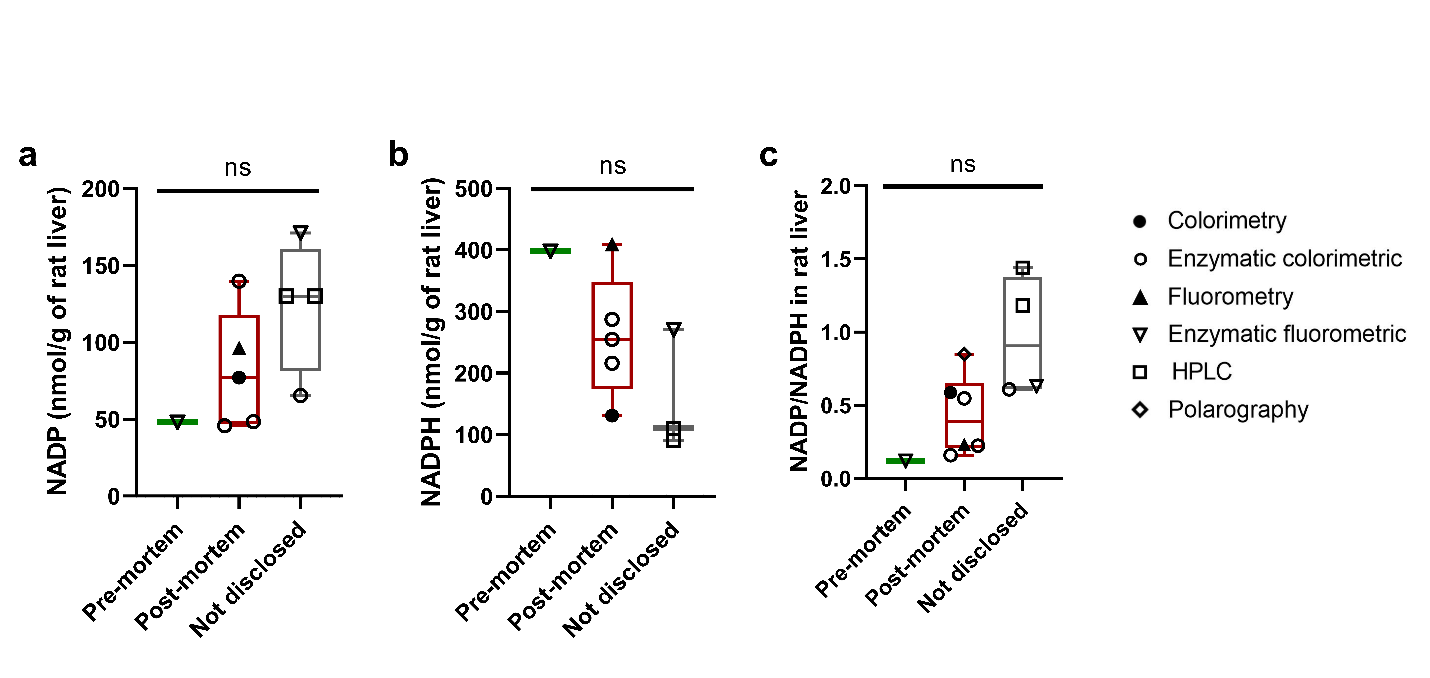
**

**Supplementary figure 9: Effects of pre- versus post-mortem tissue collection on NADP(H) levels in normal rat liver.** a-c: Reported (a) NADP, (b) NADPH, (c) NADP/NADPH ratio in young (<18 months old) control rat liver samples harvested at different timepoints relative to sacrifice. Boxes represent 25th and 75th percentile with median line. Whiskers show min. to max. values. Statistical significance was determined by one-way ANOVA with multiple comparisons using Tukey’s test as post-hoc test.

**Supplementary figure 10: Systematic search workflow**

**
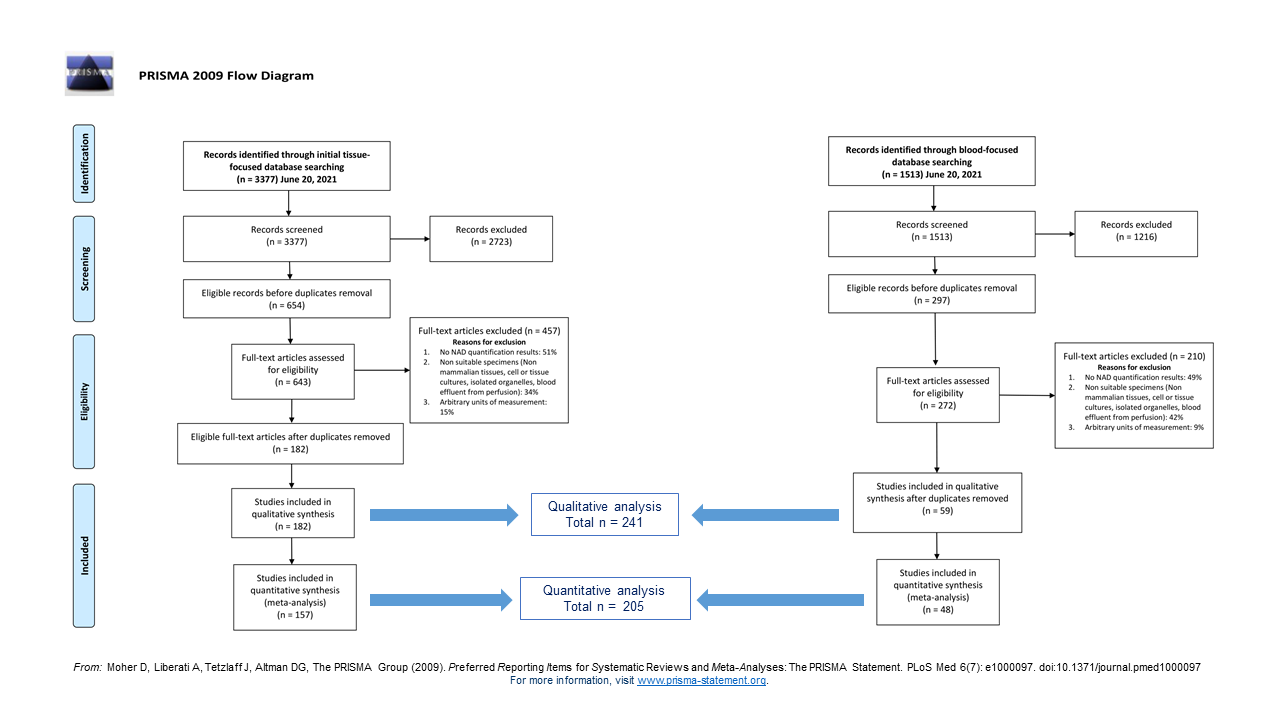
**

**Appendix S1: Medline (Ovid) tissue-focused search query.**

| 1 | NAD/ |
| --- | --- |
| 2 | nad.ti,ab,kw. |
| 3 | Nicotinamide adenine dinucleotide.ti,ab,kw. |
| 4 | 1 or 2 or 3 |
| 5 | Exp mammals/ |
| 6 | (mouse? or mice? or rat? or murine? or pig? or dog? or primate? or ape? or cat?).ti,ab,kw. |
| 7 | (human? or homo sapiens or patient).ti,ab,kw. |
| 8 | 5 or 6 or 7 |
| 9 | (sampl* adj3 (protocol* or standard* or method*)).ti,ab,kw. |
| 10 | (tissue* adj3 (sampl* or collect*)).ti,ab,kw. |
| 11 | ((animal* or human) adj3 (sampl* or tissue?)).ti,ab,kw. |
| 12 | ((muscle* or liver* or hepatic* or brain* or neural* or neuron* or kidney* or renal* or fat* or adipose) adj3 (cell* or tissue* or sample*)).ti,ab,kw. |
| 13 | 9 or 10 or 11 or 12 |
| 14 | 4 and 8 and 13 |
| 15 | Limit 14 to (english or french) |

/: subject heading, ab: abstract, ti: title , kw: keyword heading (Author keywords), exp: search for the term and any narrower subject term, ?: optional wildcard (keyword substitutions), *: focus on the term as main topic , adj3: adjacency, in any order, up to 3 words in between.

**Appendix S2: Medline (Ovid) search query focused on blood related samples.**

| 1 | NAD/ |
| --- | --- |
| 2 | nad.ti,ab,kw. |
| 3 | Nicotinamide adenine dinucleotide.ti,ab,kw. |
| 4 | 1 or 2 or 3 |
| 5 | Exp mammals/ |
| 6 | (mouse? or mice? or rat? or murine? or pig? or dog? or primate? or ape? or cat?).ti,ab,kw/ |
| 7 | (human? or homo sapiens? or patient?).ti,ab,kw. |
| 8 | 5 or 6 or 7 |
| 9 | (sampl* adj3 (protocol* or standard* or method*)).ti,ab,kw. |
| 10 | (tissue* adj3 (sampl* or collect*)).ti,ab,kw. |
| 11 | ((animal* or human* or patient*) adj3 (sampl* or tissue?)).ti,ab,kw. |
| 12 | ((Blood* or plasma* or serum* or PBMC* or PRBC* or Platelets* or lymphocytes* or leucocytes* or hematocytes*) adj3 (cell* or sample*)).ti,ab,kw. |
| 13 | 9 or 10 or 11 or 12 |
| 14 | 4 and 8 and 13 |
| 15 | Limit 14 to (english or french) |

/: subject heading, ab: abstract, ti: title , kw: keyword heading (Author keywords), exp: search for the term and any narrower subject term, ?: optional wildcard (keyword substitutions), *: focus on the term as main topic , adj3: adjacency, in any order, up to 3 words in between.
